# Sumoylation-independent activation of Calcineurin-NFAT-signaling via SUMO2 mediates cardiomyocyte hypertrophy

**DOI:** 10.1038/srep35758

**Published:** 2016-10-21

**Authors:** Alexander Bernt, Ashraf Y. Rangrez, Matthias Eden, Andreas Jungmann, Sylvia Katz, Claudia Rohr, Oliver J. Müller, Hugo A. Katus, Samuel T. Sossalla, Tatjana Williams, Oliver Ritter, Derk Frank, Norbert Frey

**Affiliations:** 1 Dept of Internal Medicine III (Cardiology and Angiology) UKSH, Campus Kiel, Germany; 2 DZHK (German Centre for Cardiovascular Research), Partner Site Hamburg-Kiel-Lübeck, Kiel, Germany; 3 Dept of Internal Medicine III, University of Heidelberg, Germany; 4 Dept of Internal Medicine I (Cardiology), University Hospital of Würzburg, Germany

## Abstract

The objective of this study was to identify unknown modulators of Calcineurin (Cn)-NFAT signaling. Measurement of NFAT reporter driven luciferase activity was therefore utilized to screen a human cardiac cDNA-library (~10^7^ primary clones) in C2C12 cells through serial dilutions until single clones could be identified. This extensive screening strategy culminated in the identification of SUMO2 as a most efficient Cn-NFAT activator. SUMO2-mediated activation of Cn-NFAT signaling in cardiomyocytes translated into a hypertrophic phenotype. Prohypertrophic effects were also observed in mice expressing SUMO2 in the heart using AAV9 (Adeno-associated virus), complementing the *in vitro* findings. In addition, increased SUMO2-mediated sumoylation in human cardiomyopathy patients and in mouse models of cardiomyopathy were observed. To decipher the underlying mechanism, we generated a sumoylation-deficient SUMO2 mutant (ΔGG). Surprisingly, ΔGG replicated Cn-NFAT-activation and the prohypertrophic effects of native SUMO2, both *in vitro* and *in vivo*, suggesting a sumoylation-independent mechanism. Finally, we discerned a direct interaction between SUMO2 and CnA, which promotes CnA nuclear localization. In conclusion, we identified SUMO2 as a novel activator of Cn-NFAT signaling in cardiomyocytes. In broader terms, these findings reveal an unexpected role for SUMO2 in cardiac hypertrophy and cardiomyopathy, which may open the possibility for therapeutic manipulation of this pathway.

The heart reacts to any pathological biomechanical stress typically by developing myocardial hypertrophy. After an initial compensatory phase, hypertrophic growth of the myocardium as well as further remodeling eventually causes dilated cardiomyopathy, heart failure, arrhythmias, and sudden death. A broad network of signaling pathways controls physiological and pathophysiological cardiomyocyte growth^1^. One of the key signaling pathways associated with cardiac hypertrophy is mediated by calcineurin (Cn), a calcium-calmodulin dependent serine-threonine protein phosphatase, that has been identified as pro-hypertrophic signaling modulator more than a decade ago^2^. Once activated, calcineurin dephosphorylates N-terminal serine residues on cytoplasmic transcription factors of the NFAT family, leading to nuclear translocation of NFATs, enabling them, in concert with other transcription factors such as MEF2 or GATA1, to activate a prohypertrophic set of genes^3^,^4^,^5^. Interestingly, it has been shown that a subpool of calcineurin itself also translocates to the nucleus^6^, implying the existence of additional targets for dephosphorylation. Activation of the Cn-NFAT pathway in transgenic mice overexpressing a constitutively active mutant of Cn causes dramatic cardiac hypertrophy with severe fibrosis and activation of the molecular hypertrophic program^2^. Moreover, cardiomyocytes from these mice hearts are disorganized and hypertrophic with doubled cross-sectional area compared to wild type cardiomyocytes. To the contrary, inhibition of Cn-NFAT signaling, observed e.g. in *calcineurin Aβ*−/−, *Nfatc3*−/− and *Nfatc2*−/− mice, led to the inhibition of pathological cardiac hypertrophy in response to pressure overload or stimulation with neuroendocrine agonist infusion^2^,^7^,^8^. Together, these findings demonstrate a critical and indispensable role Cn-NFAT signaling plays in the pathological remodeling of the heart.

Small ubiquitin related modifiers (SUMO) are a family of four different proteins (human SUMO1–4) that can covalently modify their target proteins by reversible conjugation, thereby influencing protein activity, localization and stability^9^,^10^,^11^. Sumoylation has been associated to a variety of different cellular processes including cell cycle regulation, transcription and subcellular transport^12^ via alterations in molecular interaction patterns of the modified target (reviewed in ref. 13). SUMO proteins resemble the three-dimensional structure of ubiquitin but share only about 20% amino-acid sequence^14^,^15^,^16^. SUMO is expressed as a precursor protein that has to be cleaved in order to be activated through accessibility of two C-terminal glycines (SUMO diglycine motif). SUMO2 and −3 share 97% sequence identity but only 50% with SUMO1, resulting in distinct functions for the different small modifiers^17^. Of note, SUMO2 among other SUMO family members has been implicated in cardiac disease as extensively reviewed in refs 18, 19, 20, 21. Apart from the well-studied sumoylation modifications, there has been a recent discovery of non-covalent sumo-interaction or –binding motifs (SIM/SBM)^22^,^23^ which influence their targets, all of which are themselves sumoylated proteins^23^,^24^,^25^,^26^.

The purpose of the present study was to identify yet unknown modulators of calcineurin-NFAT signaling in the heart. We found and confirmed SUMO2 to be a novel and potent activator of this signaling pathway. We further show that SUMO2 activates signaling by direct interaction with calcineurin A. Cn activation is triggered by SUMO2-overexpression and inhibited by SUMO2-knockdown in either skeletal muscle cells or neonatal rat ventricular cardiomyocytes (NRVCM). SUMO2 overexpression yields higher expression levels of hypertrophy-associated genes and significantly increased cell surface area. Finally, we found strong evidence for SUMO2 activating calcineurin and cardiomyocyte hypertrophy via a sumoylation-independent mechanism, uncovering a novel pathway in the regulation of calcineurin/NFAT activity.

## Methods

### Cloning of full length and ΔGG-deletion of SUMO2

Full-length and ΔGG mutant of mouse *sumo2* were cloned from mouse heart cDNA by using primers listed in Table S1 for expression in NRVCM.

### Generation of recombinant adenoviruses for recombinant protein expression

An adenovirus (Ad) encoding the full length mouse *sumo*2 and *sumo2*ΔGG-construct and other necessary constructs (microRNA against *sumo2*) were generated using the ViraPower™ Adenoviral Kit (Life Technologies, Karlsruhe, Germany) according to the manufacturer’s instructions. Titration of the virus was carried out by staining infected HEK293A cells with FITC-labeled anti-Hexon antibody. NRVCM were infected with a multitude of infection (moi/ifu) of 50 moi, or otherwise indicated. Similarly, for the generation of expression plasmids, vectors were shuttled into pcDNA-Dest40 or pcDNA3.2.

### Protein preparation

NRVCM were lysed by 3 freeze-thaw cycles in lysis buffer. Cell debris was removed by centrifugation at 12,000 × g for 20 minutes and protein concentration was determined photometrically by Biorad DC-assay method (Biorad, Munich, Germany).

### Subcellular fractionation

NRVCM were fractionated using the REAP-method of subcellular fractionation^27^. Method-integrity was verified by controlling the fractions for Histone H3 (Cell signalling, #4499) and GAPDH (Sigma, #G8795) proteins.

### Immunoblotting

Protein samples were resolved by 10% SDS-PAGE, transferred to a polyvinylidenefluoride membrane and immunoblotted. After two hours of blocking in 5% dry-milk in TBS-T, Primary antibody was applied overnight at 4 °C followed by incubation with a suitable HRP-coupled secondary antibody (1:10000) (Santa Cruz, Heidelberg, Germany). Quantitative densitometric analysis was carried out with ImageJ/Fiji version 1.46.

### RNA isolation and quantitative real-time PCR

Total RNA was isolated from cultured cells using QIAzol lysis reagent (Qiagen, Germany) following the manufacturer’s instructions. One μg of DNA-free total RNA was transcribed into cDNA using the Superscript III first strand cDNA synthesis kit (Life Technologies, Darmstadt, Germany). For qRT-PCR, the EXPRESS SYBR GreenER Reagent (Life Technologies, Darmstadt, Germany) was used in CFX96 real-time Cycler (Biorad, Munich, Germany).

### Reporter gene assays

All the reporter gene assays shown in this work were performed either in NRVCM, ARVCM or C2C12-myoblasts. NRVCM were infected with combinations of different viruses expressing SUMO2 (50 moi), SUMO2ΔGG (50 moi), ΔCnA (50 moi), and LacZ as control or a filler virus to maintain equal virus load, along with adenovirus NFAT-reporter-luc (10 moi) carrying a firefly luciferase and AdRen-luc carrying renilla luciferase (5 moi, for normalization of the measurements). For SUMO2 knockdown experiments, NRVCM were infected with an Adenovirus containing a synthetic microRNA against SUMO2 or a non-targeting control micro RNA termed miR Neg. C2C12 cells were transfected with siRNA against SUMO2 (Ambion, #4390816) or a control-siRNA (control siRNA-A, Santa Cruz). Studies were performed using a dual luciferase reporter assay (Promega, Mannheim, Germany) according to the manufacturer’s instructions.

### Immunoprecipitation of endogenous proteins

Mouse left ventricles were homogenized using an Ultra-turrax tissue separator in RIPA lysis buffer, whereas, C2C12-Cells were lysed with 3 freeze-thaw cycles in Native lysis buffer (NLB) Approximately 4 μg of SUMO2 antibody or anti-HA antibody was allowed to interact with 1 mg protein for 4 h, to which, 50 μl of equilibrated Dynabeads were pipetted and incubated overnight. Precipitated proteins were eluted with 50 μl of 2x Laemmli buffer, 10–20 μl of which was subjected to SDS-PAGE, followed by transfer to polyvinylidenefluoride membranes and immunoblotted.

### Immunofluorescence microscopy

Cell size measurement was carried out in NRVCM which were prepared as described^28^,^29^. NRVCM were fixed with 4% paraformaldehyde, washed and blocked. Cells were then incubated with monoclonal mouse anti-α-actinin and respective AlexaFluor546-conjugated secondary antibodies.

### Cell surface area measurements

Cell size measurements were carried out as described^30^. In brief, 5 × 5 × 5 (x y z) pictures were taken in 20x magnification and merged. The cell size was measured using Keyence’s HybridCellCount software module in fluorescence intensity single-extraction mode.

### Proximity ligation assay

Fixed cells were blocked and incubated with two primary antibodies against SUMO2/3 (Abcam, 1:1000, ms) and CnA (Upstate, 1:50, rb). Species-specific probes were added and after annealing and ligation of these probes, an amplification and labeling step was performed that labels probes in close proximity with a green-emitting fluorophore. Green dots within a cell indicate less than 30–40 nm distance between proteins and thus imply a direct protein-protein interaction. Quantitative analysis was carried out utilizing Keyence’s Hybrid Cell Count module as described in detail in supplementary methods. In brief, nuclei were counted and PLA-fluorescence signal extracted to calculate the percentage of positively stained nuclei.

### Generation of AAV9-vectors

The cDNA sequences of SUMO2 and SUMO2ΔGG were cloned into a single-stranded AAV vector backbone as described before^31^. In Brief, after ligation of the digested ORF amplicon into the AAV genome plasmid (digested with the same enzyme), correct fragment size and orientation were controlled by agarose gel-electrophoresis and sequencing, resulting in pSSV9-CMV-MLC1500-SUMO2 or SUMO2∆GG. AAV9 vectors were generated by cotransfection of helper plasmid pDP9rs and either pSSV9-CMV-MLC1500-SUMO2, -SUMO2∆GG or -luciferase (control). AAV9 vectors were then purified and Genomic titers were determined by qRT–PCR (Schinkel *et al.*, 2006; Werfel *et al.*, 2012).

### Animal experiments

All animal experiments were approved and carried out in strict accordance to the ethical guidelines by the Ministry of Energy, Agriculture, the Environment and Rural Areas (MELUR).

### AAV9 mediated gene-transfer

Animals were injected with up to 10^12^ vector genomes per animal in a total of 120 μL PBS into the tail vein of 8 weeks old male C57BL/6N mice. Six weeks later, the animals were sacrificed after echocardiography to harvest the organs.

### Echocardiography

Echocardiographic analyses were carried out using a VisualSonics Vevo 1100 high frequency ultrasound system. In brief, mice were anesthetized using Isoflurane (2.5 ppm) and placed onto a warming pad with continuous temperature management. EF measurements were carried out by LV trace protocol comparing enddiastolic and endsystolic LV Volume. The following M-mode based parameters were obtained in a short axis on the level of the papillary muscles.

## Results

### Screening for Calcineurin-NFAT activators and validation in C2C12 cells

**Figure 1:**
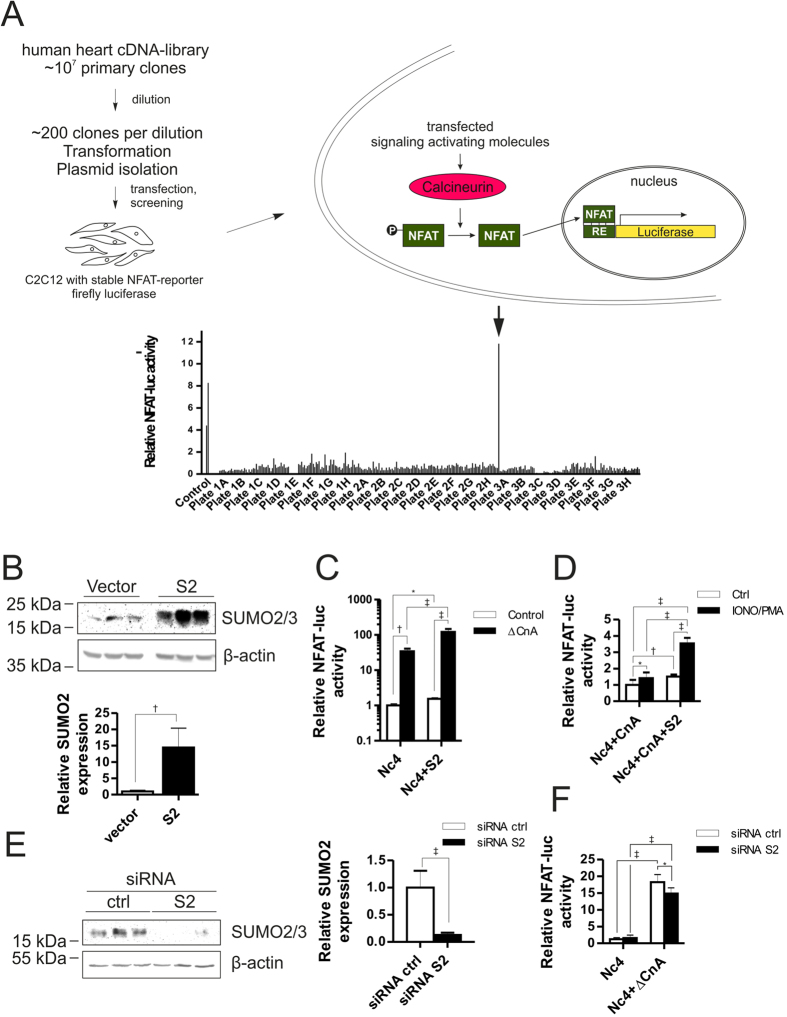
Screening for Calcineurin-NFAT activators and validation in C2C12 cells. (**A**) Starting from 10^7^ cDNA-clones from fetal human heart, we performed serial dilutions and plasmid preparations for transfection of C2C12 cells, stably carrying NFAT-reporter firefly luciferase. Pools of approximately 200 cDNAs were transfected and tested for Cn-NFAT signaling activation through luciferase activity screening. Further dilutions and plasmid preparations were carried out until single clones could be transfected and screened, subsequently sequencing Cn-NFAT activating clones. (**B**) Western blot showing SUMO2 (S2) overexpression and relative densitometry was calculated through β-actin loading control. (**C**) NFAT-responsive element driven firefly luciferase activity of S2 in the presence of NFATc4. Constitutively active calcineurin (ΔCnA) is either or not transfected in the presence or absence of S2. Shown is the mean of three independent experiments in quadruplicates. (**D**) NFAT-responsive element driven firefly luciferase activity of S2 in the presence of wild-type CnA and NFATc4. CnA is either or not activated by Ionomycin (2 μM) + PMA (1 μM) addition. Shown is the mean of three independent experiments in quadruplicates. (**E**) Western blot showing knockdown of S2 and relative densitometry was calculated through β-actin loading control. (**F**) NFAT-RE firefly luciferase activity in the presence of NFATc4 and in the presence or absence of ΔCnA and/or siRNA against *sumo2* or control siRNA. Shown is the mean of three independent experiments in quadruplicates. All experiments were performed in C2C12 cells stably expressing NFAT-RE driven firefly luciferase. Statistical calculations were carried out by two-tailed Student’s t-test (**B**,**E**) or Two-way ANOVA (**C**,**D**,**F**). *p < 0.05, ^†^p < 0.01, ^‡^p < 0.001.

In order to identify unknown activators of calcineurin-NFAT signaling we devised a cDNA screening strategy, utilizing a commercially available fetal human heart cDNA-library with approximately 10^7^ primary clones. The human heart cDNA library was diluted and subdivided to yield individual pools of approximately 200 clones each, which were transformed and amplified. The plasmids were then isolated and transfected in C2C12 myoblast cells stably carrying an NFAT-reporter driven firefly luciferase gene in a 12-well format (Fig. 1A). We selected a cutoff for further investigating cDNA-pools that show increased luciferase activity and carried on with one pool of cDNAs that showed higher luciferase activity than the positive controls (Fig. 1A). This pool was then further diluted to contain approximately 12 cDNA constructs per dilution. After transformation and plasmid preparation, these pools were re-transfected into the C2C12 cells in 24-well plates to determine the NFAT-signaling activation. After one additional round of dilutions, single clones were transfected and positives were sequenced to identify SUMO2 as the strongest activator of Cn-NFAT signaling pathway.

To further validate the data from the screening experiment, we generated a SUMO2 (S2) wild-type construct for expression in C2C12 cells and validated its overexpression both at protein (Fig. 1B) and RNA-level (Figure S1A). We reconfirmed the activation of NFAT-signaling by transfecting C2C12 cells with NFATc4 or SUMO2 in the presence or absence of constitutively active calcineurin A (ΔCnA) (Figure S1B) and observed a strong activation of NFAT-signaling through ΔCnA as well as a significant additive effect of SUMO2 on NFAT-signaling in the presence of ΔCnA (Fig. 1C). These results were highly reproducible in HEK cells, a human derived cell line (Figure S1D). A similar result was observed by transfecting C2C12 cells with wild-type calcineurin A (CnA) and activating it through addition of Ionomycin and PMA (Fig. 1D). We next investigated the effect of SUMO2 downregulation on calcineurin and NFAT-signaling via siRNA mediated knockdown of SUMO2 in C2C12 cells. Transfection of these cells with a SUMO2 siRNA led to 85% knockdown at protein- and 78% at RNA level, respectively (Fig. 1E and Figure S1C). Although ΔCnA still increased the NFAT-signaling activity it was significantly reduced upon knockdown of SUMO2 compared to the control-siRNA transfections (Fig. 1F).

### SUMO2 activates Calcineurin-NFAT signaling in NRVCM

**Figure 2:**
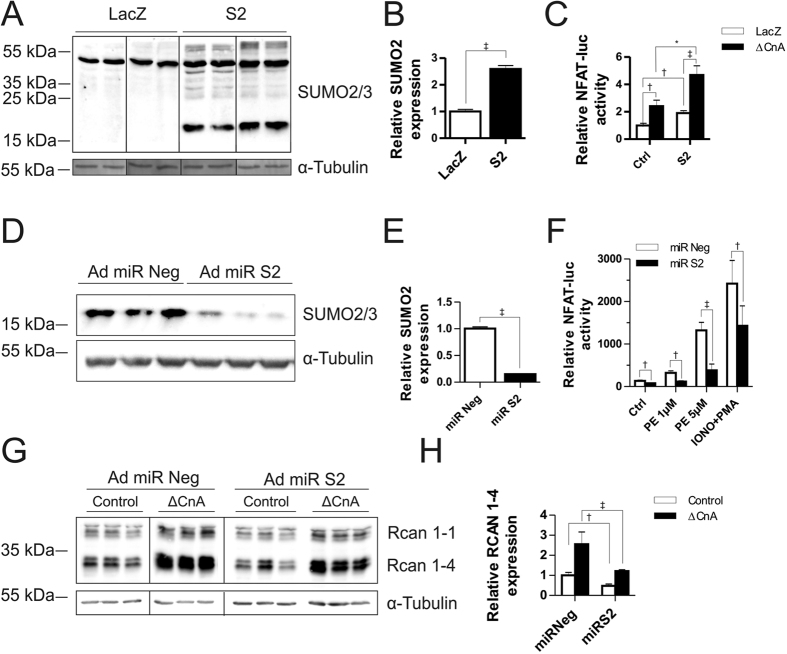
SUMO2 induces hypertrophy in NRVCM. (**A**) Western blot showing S2 overexpression in the presence or absence of adenoviral (Ad) S2 overexpression and relative densitometry was calculated through α-tubulin loading control (**B**). (**C**) NFAT-RE firefly luciferase construct was expressed via adenoviral infection and luciferase activity measured in the presence or absence of ΔCnA and S2. Shown is the mean of three independent experiments in hexaplicates. (**D**) Western blot showing S2 knockdown with microRNA against *sumo2* (Ad miR S2) compared to negative control microRNA (Ad miR Neg) and relative densitometry was calculated through α-tubulin loading control (**E**). (**F**) Ad NFAT-RE firefly luciferase activity in the presence of miR S2 (96 h knockdown) compared to miR Neg in the presence of PE (48 h treatment at 1 μM or 5 μM) and Ionomycin/PMA (6 h treatment at 2 μM/4.5 nM). Data represented as a mean of three independent experiments performed in hexaplicates. (**G**) Western blot showing 96 h SUMO2 knockdown (Ad miR S2) compared to negative control (Ad miR Neg) in the presence or absence of constitutively active calcineurin A (ΔCnA). (**H**) Relative densitometry was calculated through α-tubulin loading control. All experiments were performed in NRVCM cells. Statistical calculations were carried out by two-tailed Student’s t-test (**B**,**E**) or Two-way ANOVA (**C**,**F**,**G**). *p < 0.05, ^†^p < 0.01, ^‡^p < 0.001. Dividing lines represent rearrangements of respective lanes within one membrane.

Through our luciferase screen, we identified SUMO2 as a robust activator of NFAT-signaling in C2C12 cells, a mouse skeletal myoblast cell line. However, we observed ubiquitous expression of *sumo2* in various tissues including lung, liver, kidney, etc. (Figure S2A). Moreover, *sumo2* expression was relatively higher in the heart compared to the skeletal muscle. Therefore, we next studied if SUMO2 overexpression also affects cardiac NFAT-signaling. We generated an adenoviral mammalian expression construct for SUMO2 to be used in NRVCM, which expressed recombinant protein at significant levels (Fig. 2A,B, Figure S2B). As observed in C2C12 cells, overexpression of SUMO2 led to activation of NFAT-signaling in NRVCM (Fig. 2C, Figure S2C). Similarly, the activator effect of S2 was consistently observed in adult rat ventricular cardiomyocytes (ARVCM) as overexpression of S2 caused significant activation of NFAT-signaling at baseline, which further increased in the presence of ∆CnA (Figure S2D). A synthetic microRNA-mediated knockdown of SUMO2 (Fig. 2D,E) substantially inhibited NFAT-signaling, even in the presence of NFAT-signaling activators, phenylephrine (PE) or Ionomycin and PMA (Fig. 2F). Consistently, NFAT activation through constitutively active calcineurin A strongly increased the transcript levels of Rcan1–4, an exquisitely NFAT-sensitive gene, which is abrogated when SUMO2 is knocked down (Fig. 2G,H).

### SUMO2 induces hypertrophy in NRVCM

**Figure 3:**
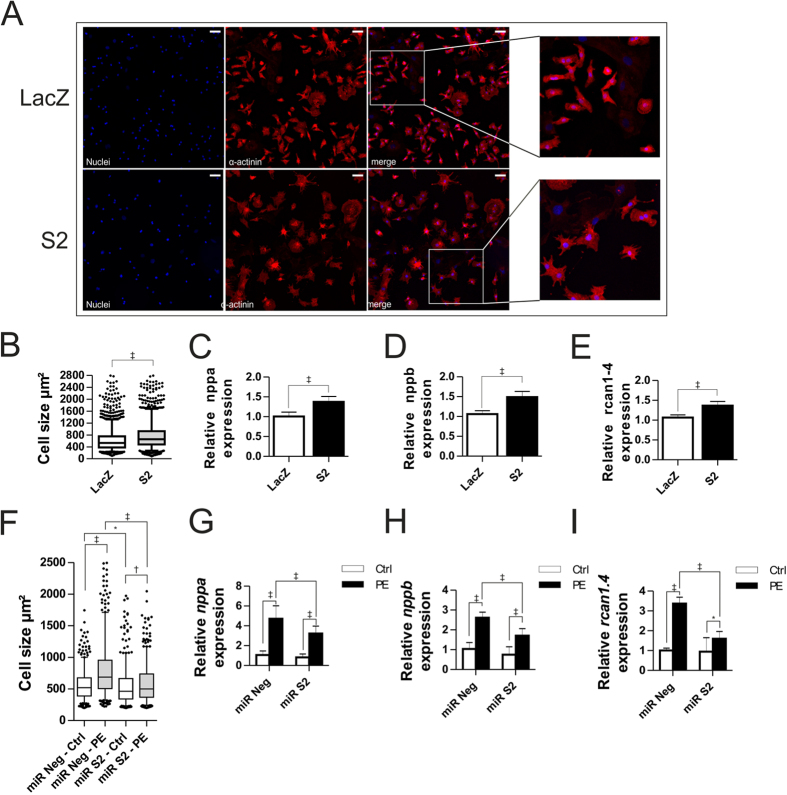
S2 induces hypertrophy in NRVCM. (**A**) Representative immunofluorescence analysis of fixed NRVCM-cells overexpressing LacZ or S2 (scale bar 50 μm). (**B**) Cell surface area of NRVCM. Shown are the analyses of three independent experiments in triplicates with n > 1000 counted cells per replicate. (**C**–**E**) mRNA expression levels of *nppa*, *nppb and rcan1–4* respectively in the presence or absence of AdS2. Shown is the mean of three independent experiments in hexaplicates. (**F**) Cell surface area in the presence or absence of 5 μM PE after 72 h of control- (miR Neg) or SUMO2-knockdown conditions (miR S2). (**G**–**I**) mRNA expression levels of *nppa*, *nppb and rcan1-4* respectively in the presence or absence of 5 μM PE under 72 h of control (miR Neg) or SUMO2-knockdown conditions (miR S2). Shown is a mean of three independent experiments in quadruplicates. All experiments were performed in NRVCM cells. Statistical calculations were carried out by two-tailed Student’s t-test (**C**–**E**), Wilcoxon rank-sum test (**B**), non-parametric two-way-ANOVA by ranks (Friedman test, (**F)**), or two-way-ANOVA (**G–I**). *p < 0.05, ^†^p < 0.01, ^‡^p < 0.001.

Cn-NFAT signaling is one of the key signaling pathways directly involved in the induction of pathological cardiac hypertrophy. In order to assess the pathophysiological relevance of SUMO2 in cardiac disease, we studied the SUMO2-dependent sumoylation status in two different mouse models of cardiac disease, calcineurin transgenic and TAC (Transverse Aortic Constriction) operated mice. Cn-transgenic mice show pronounced hypertrophy followed by heart failure, whereas, TAC operated mice suffer from pressure overload, resulting in hypertrophy and heart failure. Both models displayed a significant increase in native monomeric SUMO2 compared to respective control mice (Figure S3A,B,D,E). While SUMO2 is involved in many pathways and its covalent attachment to other proteins is therefore expected to be differentially regulated, both disease models showed an overall increase in protein sumoylation, and this increase was more prominent in calcineurin transgenic mice (Figure S3A,C,D,F). A strong increase in SUMO2-mediated sumoylation was also observed in the myocardium of human patients suffering of dilated (DCM) or ischemic cardiomyopathy (ICM), implying an association of SUMO2 with human disease (Figure S3G,H). Given the strong activation of NFAT-signaling in NRVCM, C2C12 and HEK cells, and the upregulation of SUMO2 in mouse models of cardiac hypertrophy as well as in human patients suffering from DCM or ICM, we investigated the potential phenotypic and molecular effects of SUMO2 in NRVCM. Adenoviral overexpression of SUMO2 led to a significant increase in NRVCM surface area compared to LacZ control virus infected cells (Fig. 3A,B). In line with this finding, we observed a significant upregulation of the fetal genes *nppa* and *nppb* (Fig. 3C,D). Moreover, the expression of *rcan1–4,* a known NFAT-responsive gene^32^,^33^, was increased upon SUMO2 overexpression (Fig. 3E). Conversely, miRNA mediated knockdown of SUMO2 significantly reduced the cell-size at basal level, as well as it abrogated the prohypertrophic effects of PE (Fig. 3F). Along the same lines, expression of *nppa, nppb* and *rcan1–4* was significantly upregulated in PE-treated cells, whereas, this increase was attenuated in cells upon SUMO2 knockdown conditions (Fig. 3G–I). Taken together, these data indicate that SUMO2 overexpression induces cardiomyocyte hypertrophy, consistent with the robust activation of Cn-NFAT signaling *in vitro*.

### SUMO2 mediated activation of calcineurin-signaling and cellular hypertrophy are sumoylation independent

**Figure 4:**
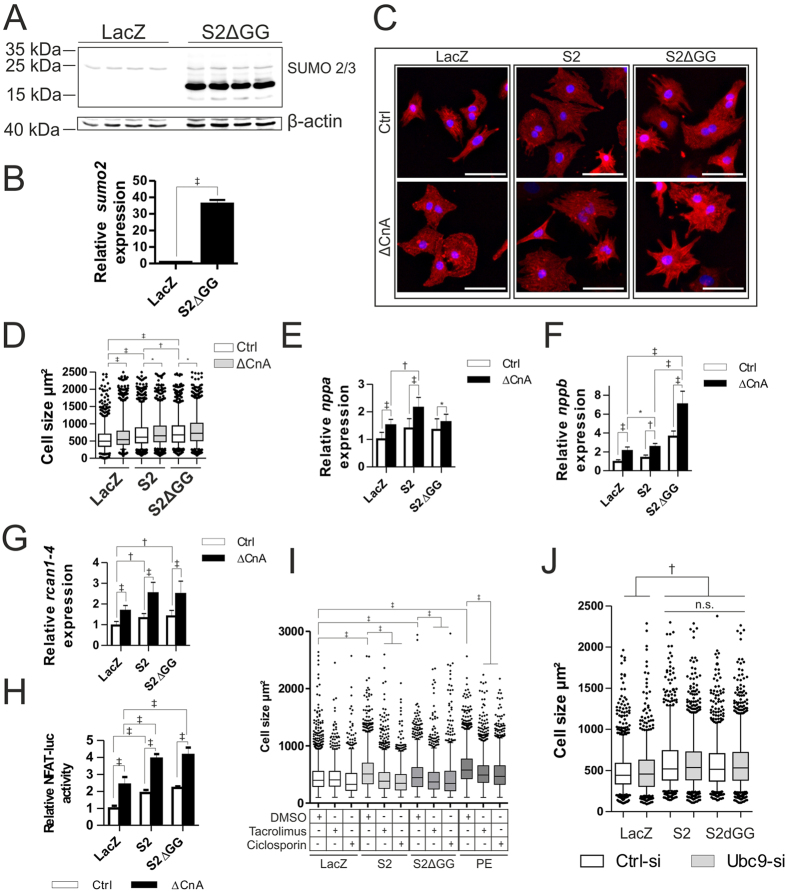
SUMO2 mediated activation of calcineurin-signaling and cellular hypertrophy are sumoylation independent. (**A**) Western blot showing overexpression of S2ΔGG compared to LacZ. (**B**) mRNA levels of *sumo2* in the presence or absence of overexpressed S2ΔGG. Shown is the mean of three independent experiments in quadruplicates. (**C**) Representative immunofluorescence analysis of fixed NRVCM cells in the presence or absence of overexpressed ΔCnA along with overexpression of LacZ (control), S2 or S2ΔGG (scale bar 50 μm). (**D**) Respective cell surface area. Shown is the analysis of three independent experiments with n > 1000 cells per replicate. (**E**–**G**) mRNA expression levels of *nppa*, *nppb and rcan1-4,* respectively, in the presence or absence of overexpressed ΔCnA along with overexpressed LacZ (control), S2 or S2ΔGG. For (**E**–**G**) Shown is the mean of three independent experiments in hexaplicates. (**H**) NFAT-RE firefly luciferase activity in the presence of either S2 or S2ΔGG and in the presence or absence of ΔCnA compared to LacZ control. Shown is the mean of three independent experiments in hexaplicates. (**I**) Cell surface area in the presence or absence of overexpressed S2 or S2ΔGG compared to LacZ negative-control or PE (5 μM, positive-control) in the presence or absence of either Tacrolimus or Ciclosporin. Shown are the analyses of three independent experiments in triplicates with n > 1000 counted cells per replicate. (**J**) Cell surface area with overexpressed LacZ, S2 or S2ΔGG in the presence or absence of UBC9-knockdown. All experiments were performed in NRVCM cells. Statistical calculations were carried out by using two-tailed Student’s t-test (**B**), non-parametric two-way-ANOVA by ranks (Friedman test, (**D,I,J**)), or two-way-ANOVA (**E**–**H**). *p < 0.05, ^†^p < 0.01, ^‡^p < 0.001.

As sumoylation of proteins is the major mechanism of modification by which SUMO proteins exert their structural and/or functional effects, we investigated a sumoylation-deficient SUMO2 mutant to determine whether the effects observed for SUMO2 are sumoylation-dependent or -independent. We therefore created a diglycine-deficient mutant (SUMO2ΔGG) construct via site directed mutagenesis (Figure S4A) and cloned it into an adenoviral expression vector for overexpression in NRVCM (Fig. 4A,B, Figure S4B). Like SUMO2, overexpression of this mutant also induced cellular hypertrophy in NRVCM (Fig. 4C,D). Co-expression of ΔCnA with SUMO2 or SUMO2ΔGG resulted in a comparable additive increase in cell size in NRVCM compared to SUMO2 or SUMO2ΔGG alone (Fig. 4C,D, Figure S4D). ΔCnA increases the expression of *nppa, nppb* and *rcan1–4* significantly and this effect is again exaggerated by the addition of either SUMO2 or SUMO2ΔGG (Fig. 4E–G). We then repeated the NFAT-reporter mediated luciferase activity assay with SUMO2ΔGG in the presence or the absence of ΔCnA. In the presence of overexpressed ΔCnA, SUMO2ΔGG led to a strong activation of NFAT-signaling in NRVCM, comparable to overexpressed wildtype SUMO2 (Fig. 4H, Figure S4C). Given the same impact of overexpressed SUMO2 and SUMO2ΔGG on NFAT-signaling in the presence of ΔCnA in NRVCM, we hypothesized a sumoylation independent functional interaction between SUMO2 and calcineurin A that induces cardiomyocyte hypertrophy. When endogenous calcineurin A is inhibited by either ciclosporin or tacrolimus, the increase in cell size in the presence of overexpressed SUMO2 or SUMO2ΔGG is abolished, consistent with the notion that SUMO2 acts via calcineurin A (Fig. 4I). Lastly, to confirm a sumoylation-independent mechanism, we established an si-RNA mediated knockdown of UBC9, the only E2-enzyme for SUMO2 which is essential for the ligation of SUMO2 to target proteins (Figure S4E,F). We found that the knockdown of UBC9 neither altered the cell size under control conditions, nor did it abrogate the increase in cell-size mediated by SUMO2, or SUMO2ΔGG in NRVCM (Fig. 4J). We additionally checked for SUMO2 induced alterations on SUMO1 or the ubiquitination profile and found no significant differences between control and SUMO2 expressing cells (Figure S5A,B,D,E). Moreover, overexpression of SUMO1 did not affect NFAT-reporter driven luciferase activity (Figure S5C). These observations strongly argue against the possible involvement of SUMO1 or ubiquitin in the observed SUMO2 effects.

### AAV9-mediated gene transfer of SUMO2 or SUMO2ΔGG causes cardiac hypertrophy in mice

**Figure 5:**
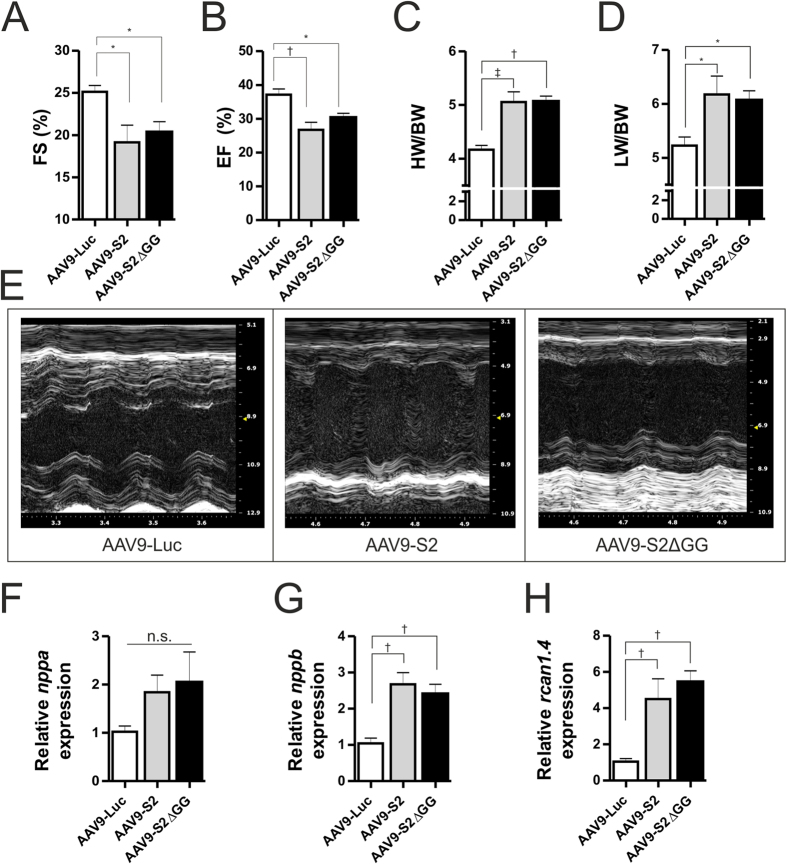
AAV9-mediated gene transfer of S2 or S2ΔGG causes cardiac hypertrophy in mice. Echocardiographic analyses at endpoint: (**A**) Fractional shortening in % (M-Mode); (**B**) ejection-fraction in % (B-Mode). (**C**) Heart weight (mg) to bodyweight (g) ratio; (**D**) lung weight (mg) to bodyweight (g) ratio. (**E**) Representative echocardiography images (M-Mode). (**F**–**H**) mRNA expression levels of *nppa*, *nppb and rcan1-4* respectively. All data obtained in the presence of AAV9-mediated overexpression of S2 or S2ΔGG, compared to Luciferase control. Mean of n = 5 mince per group, 14 weeks old, injection of AAV9 at 8 weeks. Statistical calculations were carried out by One-way ANOVA with Student-Newman-Keuls post-hoc test (**A**–**J**). *p < 0.05, ^†^p < 0.01, ^‡^p < 0.001.

To verify a (patho-) physiological relevance for the observed prohypertrophic effects of SUMO2 *in vivo*, we applied an adeno-associated-virus 9 (AAV9)-mediated gene transfer approach in mice. Therefore, 8 weeks old C57BL/6N mice were injected with up to 10^12^ vector-genomes per animal for the overexpression of SUMO2 or SUMO2ΔGG and morphological and functional data was acquired 6 weeks post-injection. Mice showed enhanced expression of S2 in the left ventricles (Figure S6A,B) as well as a good specificity towards expression in the heart (Figure S6C,D). The predominant expression is achieved in the left and right ventricle, whereas, a moderate off-target expression was seen in liver, kidney, musculus soleus and musculus gastrocnemicus compared to the luciferase control group. Functionally, mice overexpressing SUMO2 or SUMO2ΔGG displayed a significant decrease in fractional shortening (SUMO2: −24%, SUMO2ΔGG: −21%; Fig. 5A) and ejection-fraction (SUMO2: −25%, SUMO2ΔGG: −17%; Fig. 5B,E) compared to control-mice overexpressing luciferase. We also observed a significant increase in heart to body weight (Fig. 5C) and lung to body weight ratios (Fig. 5D), suggesting the development of maladaptive hypertrophy and pulmonary congestion in these mice. The respective echocardiographic data can be examined in Table S2.

At the molecular level and in line with our *in vitro* findings, overexpression of SUMO2 or SUMO2ΔGG caused significant upregulation of the fetal gene *nppb* (Fig. 5G) and an upregulation of the calcineurin sensitive gene *rcan1–4* (Fig. 5H). Though not significant, we observed a clear trend towards an upregulation of the fetal gene *nppa* (Fig. 5F).

### SUMO2 directly interacts with CnA

**Figure 6:**
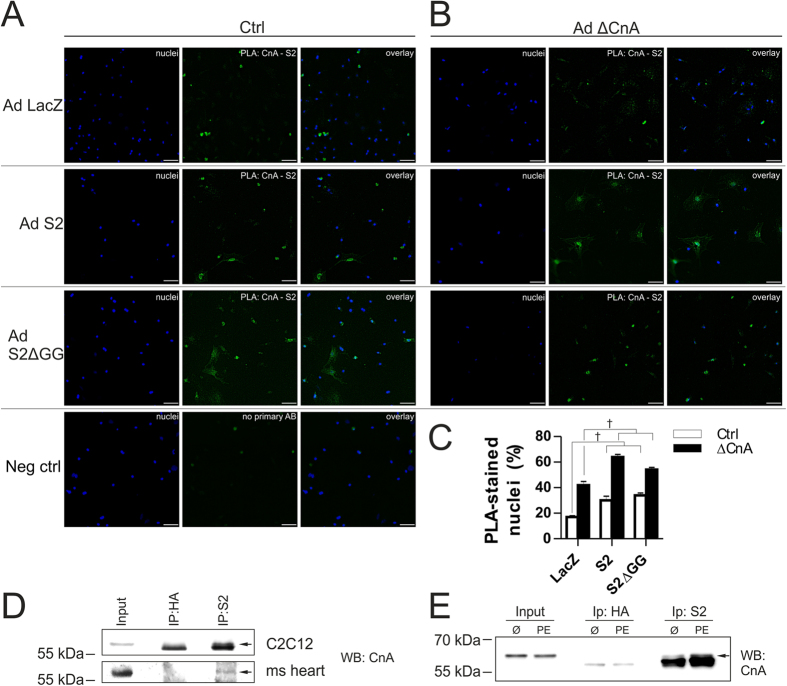
S2 directly interacts with CnA. Proximity-ligation-assay with primary antibodies against SUMO2/3 and CnA in NRVCM infected with either S2 or S2ΔGG in the presence (**A**) or absence (**B**) of ΔCnA; Negative control condition is without primary antibodies, scale bar 50 μm. (**C**) Percentage of PLA-positive stained nuclei, n = 2 per condition in three independent experiments. (**D**) Immunoprecipitation under native buffer conditions of C2C12-cell and mouse heart lysates. (**E**) Immunoprecipitation of NRVCM proteins in the presence or absence of 5 μM PE. Arrows indicate band of interest. All immunoprecipitations were performed using Dynabeads and Antibodies against HA (sigma, control) and SUMO2 + 3 (Abcam, IP) as explained in methods. 50 μg of input lysate was loaded. For Immunoblotting, CnA antibody (BD) was used.

To assess a potential interaction between SUMO2 and CnA, we performed an *in situ* proximity-ligation-assay in NRVCM overexpressing either S2 or S2ΔGG in the presence or absence of ΔCnA. Control-conditions show a co-localization between endogenous S2 and CnA as represented by green dots within the cells, where the two proteins are in closer proximity than 30–40 nm (Fig. 6A, Ad LacZ). Overexpression of S2 or S2ΔGG increased the amount of detected signals for co-localization (+14%, +17%, respectively), pointing towards an increased interaction, when cells are expressing elevated amounts of S2 (Fig. 6A,C, Ad S2, Ad S2ΔGG). These effects can be further exaggerated by co-overexpressing the interaction partner ΔCnA alongside LacZ, S2 or S2ΔGG (LacZ + ΔCnA: 41%, S2 + ΔCnA: 64%, S2ΔGG + ΔCnA: 54%, Fig. 6B,C). Signals can be detected within the nucleus as well as in the cytoplasm. Additionally, we performed co-immunoprecipitation experiments of endogenous SUMO2 and calcineurin A in C2C12 cells and in mouse heart tissue. As shown in Fig. 6D, SUMO2 interacts with calcineurin A in C2C12 cells as well as in mouse heart lysate under native conditions. To examine this interaction in physiological conditions known to cause cardiomyocyte- or cardiac hypertrophy, we also performed co-immunoprecipitation experiments in samples from PE treated cells (Fig. 6E). As anticipated, hypertrophic conditions moderately enhanced the interaction between SUMO2 and CnA after PE treatment (Fig. 6E).

### SUMO2 tethers CnA to the nucleus in cardiomyocytes

**Figure 7:**
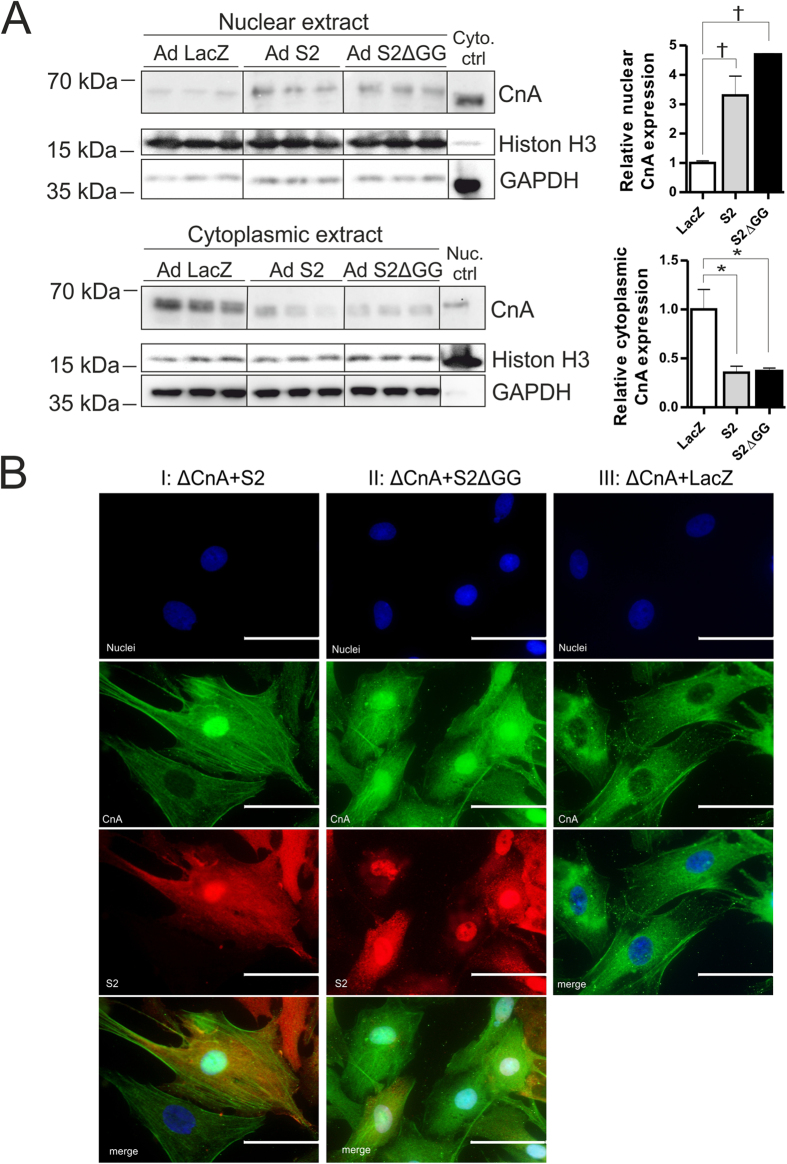
S2 tethers CnA to the nucleus in cardiomyocytes. (**A**) Western blot analysis of cytoplasmic and nuclear fractions of NRVCM with overexpressed LacZ, S2 or S2ΔGG with respective densitometric analyses, GAPDH and Histone H3 were utilized as normalization and cross-contamination controls, vertical bars within the blots represent cropped sections from the same blot. (**B**) Representative immunofluorescence analysis of fixed NRVCM cells overexpressing ΔCnA in combination with S2 (I), S2ΔGG (II), or alone (III); CnA, green; S2 and S2ΔGG, red; DAPI, blue, scale bar 50 μm. Statistical calculations were carried out by Two-way-ANOVA, *p < 0.05, ^†^p < 0.01.

Calcineurin activation has previously been linked to its nuclear translocation. Therefore, we analyzed the localization of CnA within the cell in the presence or absence of SUMO2 variants. We used adenoviral constructs for SUMO2 and SUMO2ΔGG overexpression to perform subcellular localization analyses. We observed an increase in the nuclear CnA pool when SUMO2 is overexpressed in NRVCM (Fig. 7A). The amount of cytoplasmic calcineurin is reduced at the same time (Fig. 7A). In agreement with the data shown above, this SUMO2 mediated effect on CnA is sumoylation-independent, since SUMO2ΔGG also induced activation and subsequent translocation of CnA to the nucleus, without any apparent difference compared to wild-type SUMO2 (Fig. 7A). Additionally we performed immunofluorescence analyses to revisit the subcellular localization of CnA by overexpressing SUMO2, SUMO2ΔGG and ΔCnA, in various combinations. As shown in Fig. 7B, CnA was confirmed to shuttle into the nucleus in NRVCM overexpressing ΔCnA and SUMO2 (I, rows 2 and 3) or SUMO2ΔGG (II, rows 2 and 3), whereas it remained cytoplasmic in cells overexpressing ΔCnA alone (I, rows 2 and 3, III, row 2).

## Discussion

Cn-NFAT signaling is one of the crucial cardiac signaling pathways causing the development of pathological hypertrophy upon increased biomechanical stress, e.g., remodeling following myocardial infarction, chronic hypertension or valvular heart disease^34^,^35^,^36^. Though extensive knowledge has been accumulated over the last 15 years about the role calcineurin plays in the heart, there are still very few direct calcineurin activators known to date, besides the upstream calcium-calmodulin-axis. Therefore, in the present study we aimed to identify alternative modulators of calcineurin-NFAT signaling and their influence on the development of cardiomyocyte hypertrophy. We found SUMO2 to be a strong activator of this signaling pathway in four different cell types, C2C12 cells, a mouse skeletal myoblast cell line, in ARVCM and NRVCM, primary cultured cells from adult or neonatal rat ventricles as well as in human-derived HEK-cells. The effects were first measured via NFAT-driven luciferase reporter activity and were very similar among the four different cell types. Moreover, we discovered that SUMO2 induces cardiomyocyte hypertrophy in NRVCM and mice, and does so in a sumoylation independent manner via a direct interaction with calcineurin A.

Luciferase mediated screening for modulators of specific proteins or signaling pathways led to the identification of novel protein interactions in the past^37^,^38^. Similar to a screening experiment Chang *et al.* performed for the identification of HDAC class II modulators^37^, we designed a screening strategy based on NFAT-reporter mediated firefly luciferase assay in search for novel modulators of calcineurin-NFAT signaling. Therefore, serial dilutions of a human heart cDNA library were transfected and expressed in the presence of NFAT-reporter luciferase and calcineurin that led to the identification of a potential activator of this signaling pathway, SUMO2. Considerable research efforts have earlier been undertaken on endogenous modulators of calcineurin activity, their majority yielding inhibitory molecules^4^,^39^. Cn is activated by a rise of intracellular Ca^2+^ levels. Ca^2+^ binds to calmodulin, which in turn induces a conformational change of calcineurin associated with displacement of its auto-inhibitory domain. We have previously reported that a protein termed LMCD1/Dyxin acts as a strong activator of calcineurin-mediated cardiac hypertrophy^40^. On the other hand, a variety of CnA inhibitors have been published. For example, Cain is a non-competitive inhibitor for Cn in neurons, whereas AKAP79 directly interacts and blocks Cn activity in cardiomyocytes. A family of calcineurin-interacting proteins, calsarcins is also crucial for the hypertrophic Cn-NFAT pathway, negatively regulating CnA activity and thus NFAT activation^41^. Consistently, calsarcin-1 knockout mice show exaggerated hypertrophy following pressure overload^42^. The pharmacologic agents ciclosporin and FK506/tacrolimus have been widely used in immunosuppressant therapy following organ transplant action and were only later found to inhibit calcineurin by binding to endogenous immunophilin proteins, cyclophilin A and FKBP12, respectively^43^. Rcan1–4 is another essential calcineurin/NFAT modulator and also serves as an endogenous indicator of the activation of calcineurin-NFAT signaling^44^. Rcan1–4 is one of the target genes of NFAT, but its precise function is still under debate due to both inhibitory and activator roles which have been observed under different experimental conditions^45^,^46^,^47^,^48^. More recently however, employing a systems biology approach and using single-cell experimentation in combination with *in silico* simulations, Shin *et al.* have suggested a dose dependent effect of Rcan1–4 as an inhibitor at lower levels but as a facilitator at higher expression levels^49^.

Here, we describe SUMO2 as an activator of the Cn-NFAT signaling pathway in skeletal muscle cells, in neonatal and adult rat cardiomyocytes (NRVCM, ARVCM), in mouse heart, and in a human derived cell line. Along the same lines, knockdown of SUMO2 resulted in a significant decrease of NFAT activation, even in the presence of strong NFAT-signaling activators like phenylephrine or Ionomycin and PMA. Also, knockdown of SUMO2 abrogated PE-induced cardiomyocyte hypertrophy and profoundly inhibited the effect of PE on the expression of fetal genes *nppa and nppb* in NRVCM, suggesting an essential role for SUMO2 in PE-driven cardiomyocyte hypertrophy. Consistently, Rcan1–4 was also downregulated upon SUMO2 knockdown under conditions of constitutive CnA activation, which further supports an involvement of endogenous SUMO2 in the NFAT-Cn signaling pathway.

Studying the effect of SUMO2 in the context of cardiomyocytes is particularly interesting since small ubiquitin related modifiers have been linked to various cardiac diseases in humans. For example, Kho *et al.* suggested a role for SUMO1 in SERCA2a regulation in the context of heart failure, stabilizing the protein and its ATPase activity, whereas in failing hearts sumoylation of SERCA2a and general SUMO1 levels were greatly reduced^50^. SUMO1 was the first member of the protein family identified and has been extensively characterized. *In vivo,* AAV-mediated SUMO1 gene transfer improved cardiac function in a porcine model of heart failure^51^. Of note, SUMO2 has also been implicated in human cardiomyopathy where diminished sumoylation of Lamin A results in accelerated cell death. Lamin A is a direct SUMO2 target and shows two naturally occurring mutants associated with familial cardiomyopathy, that are localized to a sumoylation consensus-motif around Lysine^201^ 52. Very recently, Kim *et al.* demonstrated the elevation of SUMO2–3 conjugation in failing human hearts and that its cardiac-restricted expression causes cardiomyopathy in mice^21^. The authors overexpressed an activated form of SUMO2 under the control of α-MHC promoter and found varying severities of disease, relative to the expression levels of activated SUMO2, ranging from acute heart failure to chronic cardiomyopathy^21^. However, it is to be noted that the authors used an activated form of SUMO2, limiting the physiological relevance of the findings. Nevertheless, we here report the induction of cardiac hypertrophy and contractile dysfunction as well as cardiomyopathy in mice overexpressing native SUMO2 via AAV9-mediated gene transfer. Furthermore, we show that not only SUMO2-dependent sumoylation, but SUMO2 expression per se is enhanced in two important mouse models of cardiac hypertrophy and pressure overload. Cn-transgenic, as well as transverse aortic constricted mice, both show elevated levels of SUMO2 and an overall increase in sumoylated proteins compared to their wild-type littermates or sham operated mice, respectively. In line with our proposed mechanism of SUMO2 being a facilitator of Cn-NFAT signaling, especially in Cn-transgenic mice, elevated levels of SUMO2 could add to NFAT activation even though SUMO2 itself does not seem to regulate calcineurin expression. Interestingly, human patients suffering from DCM or ICM also showed a marked increase in SUMO2-sumoylated proteins, implying clinical relevance and a significant role for SUMO2 in cardiac pathophysiology.

In additional support of a facilitator effect of SUMO2 on Cn-NFAT signaling, we not only observed elevated expression levels of the hypertrophy associated genes *nppa* and *nppb* but also of the direct NFAT target *rcan1–4*. Furthermore, these molecular changes were accompanied by an increase in cell surface area when overexpressing SUMO2. This effect could be augmented by co-overexpression of constitutively active CnA, implying a synergistic functional interaction between calcineurin A and SUMO2. In support of this notion, we observed a direct interaction between CnA and SUMO2 in skeletal muscle cells, cardiomyocytes, and mouse heart. Recently, another direct interaction partner for calcineurin A has been identified, also leading to activation of the NFAT-signaling pathway. The plasma membrane Na^+^ /H^+^ -exchanger 1 was found to stimulate hypertrophic gene expression in NRVCM via calcineurin^53^.

Since the majority of SUMO-dependent modulations and modifications published so far are based on the sumoylation of target proteins, it is interesting to note that our presented mechanism of calcineurin activation is independent of sumoylation. Such sumoylation-independent mechanisms of protein activity modulation have been published before and the so-called SUMO interacting/binding motifs (SIM/SBM) are subject of research and led to a proposed consensus sequence for non-covalent SUMO-interactions^23^,^54^. There are several examples of sumoylation independent non-covalent functional SUMO1 interactions. For example, SUMO1 inhibits RAD51-mediated homologous recombination by interaction with RAD51^55^. Similarly, it protects against cell death by non-covalent interactions with Fas and tumor necrosis factor receptor 1 (TNFR1)^56^. SUMO1 can also inhibit dynamin-dependent endocytosis without a covalent modification of dynamin^57^. Lee and colleagues have shown that SUMO1 represses apoptosis signal-regulating kinase 1 (ASK-1)-activation through physical interaction and not through sumoylation^58^. Similarly, SUMO3 can co-activate EBNA2 (Epstein-Barr virus nuclear antigen 2) in the absence of direct conjugation to EBNA2^59^. SUMO3 has also been found to enhance Androgen Receptor (AR) transcription independent of sumoylation mechanism in prostate cancer cells^60^. Of note, to the best of our knowledge, SUMO2 has thus far not been implicated in any non-covalent, sumoylation-independent functional interactions.

We here demonstrate a direct, endogenous functional interaction between SUMO2 and CnA under basal or pathological stress conditions that could be correlated with SUMO2-mediated activation of calcineurin-NFAT signaling. As a possible mechanism of action of the SUMO2 induced activation of calcineurin we hypothesize a tethering of CnA to the nucleus. Nuclear translocation of calcineurin has already been reported in failing human hearts and this nuclear pool of calcineurin is important in the development of cardiac hypertrophy^6^. Although the exact role of calcineurin in the nucleus is still unknown, it has been hypothesized that it acts as a transcriptional co-activator through direct interaction with DNA-bound NFAT^61^. The inhibition of calcineurin import with an artificial import blocking peptide prevents myocardial hypertrophy^62^. With SUMO2 we have identified a protein that is directly involved in the nuclear translocation and retention of calcineurin A, suggesting that this may represent the mechanism by which SUMO2 induces cardiomyocyte hypertrophy and activation of calcineurin-NFAT signaling. This notion is strongly supported by our subcellular fractionation analyses as well as the immunofluorescence experiments. An increase in nuclear CnA seems independent of sumoylation, as the sumoylation-deficient construct S2ΔGG exerted the similar effects as those under sumoylation-competent conditions did. We also show that knockdown of UBC9 does not alter the wild-type SUMO2-mediated effects on cardiomyocyte hypertrophy, strengthening the hypothesized sumoylation-independent mechanism of action. Since UBC9 is the only E2-ligase for SUMO proteins, its knockdown can be utilized as a tool for differentiating sumoylation dependent versus independent mechanisms^13^,^63^,^64^. The understanding of posttranslational modifications like sumoylation or the protein-protein interactions through SIM/SBM seems to be of great importance for understanding the molecular mechanism leading to heart failure and cardiac hypertrophy. Modulation of these pathways could prove an effective means for altering disease development and progression in the future. We found SUMO2 as an interesting candidate for modulating the calcineurin-NFAT driven cardiac hypertrophy via sumoylation independent binding. Finally, as a proof of principle for an *in vivo* relevance of the proposed mechanism, we show that SUMO2 causes cardiac hypertrophy and contractile dysfunction in a sumoylation-independent manner *in vivo*, by means of AAV9 gene-transfer technology. Expression of genes by single-stranded AAV9 particles typically reaches its peak expression between 2 and 3 weeks after injection, resulting in an overall timeframe of 3 to 4 weeks of SUMO2 or SUMO2ΔGG overexpression in these mice. For this short period of overexpression, the observed effects support a relevant role for SUMO2 in calcineurin-driven cardiac hypertrophy and potentially cardiomyopathy in general.

In conclusion, based on an unbiased screening approach, we linked SUMO2 to increased NFAT-activation, induction of hypertrophy-associated genes, and increased cellular and cardiac hypertrophy, both, *in vitro* and *in vivo*. Moreover, we found a direct interaction between SUMO2 and CnA as well as an involvement of endogenous SUMO2 in the activation of Cn-NFAT signaling. Surprisingly, we obtained similar results for SUMO2 and sumoylation-deficient SUMO2ΔGG in every investigated parameter, which rules out a sole sumoylation-dependent effect. We thus propose a mechanism where SUMO2 causes cardiac hypertrophy independent of sumoylation by direct interaction with calcineurin A, facilitating its translocation to the nucleus. Given the relevance of the calcineurin-NFAT axis in a multitude of cells and organ systems, further investigation of this mechanism might also be important in other fields of research such as immunology and cancer biology.

## Additional Information

**How to cite this article**: Bernt, A. *et al.* Sumoylation-independent activation of Calcineurin-NFAT-signaling via SUMO2 mediates cardiomyocyte hypertrophy. *Sci. Rep.* **6**, 35758; doi: 10.1038/srep35758 (2016).

## Supplementary Material

Supplementary Information

Supplementary Movie S1

Supplementary Movie S2

Supplementary Movie S3
