# Supplementary material for: Sumoylation-independent activation of Calcineurin-NFAT-signaling via SUMO2 mediates cardiomyocyte hypertrophy
Source: Sci Rep. 2016 Oct 21;6:35758. doi: 10.1038/srep35758 (PMC5073337; doi:10.1038/srep35758)
Supplement: Supplementary Information [file srep35758-s1.pdf]

Sumoylation-independent activation of Calcineurin-NFAT-signaling via SUMO2 mediates cardiomyocyte hypertrophy

Alexander Bernt[1,2], Ashraf Y. Rangrez[1,2], Matthias Eden[1,2], Andreas Jungmann[2,3], Sylvia Katz[3], Claudia Rohr[3], Oliver J. Müller[2,3], Hugo A. Katus[2,3], Samuel T. Sossalla[1,2], Tatjana Williams[2,4], Oliver Ritter[2,4], Derk Frank[1,2,#] and Norbert Frey[1,2,#]

Supplementary Material

Supplementary Methods

Cloning of full length and ΔGG-deletion of SUMO2

Full-length and ΔGG mutant of mouse *sumo2* were cloned from mouse heart cDNA by using primers listed in Table S 1 for a first PCR, and attBFor (5'-GGGGACAAGTTTGTACAAAAAAGCTGGCACC-3') and attBRev (5'-GGGGACCACTTTGTACAAGAAAGCTGGGTCGCC-3') for a second step PCR. The PCR product was recombined into the pDonR221 plasmid (Life Technologies, Karlsruhe, Germany) using Gateway technology and then subsequently recombined into expression plasmids pcDNA-DEST40 (ΔGG-construct) and pcDNA3.1 (full length), for expression in C2C12-Cells or into pAd/CMV/V5 (all: Life Technologies, Karlsruhe, Germany) for expression in NRVCM.

Generation of recombinant adenoviruses for recombinant protein expression

An adenovirus (Ad) encoding the full length mouse *sumo*2 and *sumo2*ΔGG-construct and other necessary constructs (microRNA against *sumo2*) were generated using the ViraPower™ Adenoviral Kit (Life Technologies, Karlsruhe, Germany) according to the manufacturer's instructions. In brief, a cDNA that had been previously cloned into the pDonR221 vector was shuttled into the pAd/CMV/V5-DEST Gateway vector. PacI restriction enzyme digested pAd/CMV/V5-DEST constructs were transfected into

HEK293-A cells to produce protein expressing adenoviruses. Titration of the virus was carried out by staining infected HEK293A cells with FITC-labeled anti-Hexon antibody. A β-Galactosidase-V5 encoding adenovirus (Ad-LacZ, Life Technologies, Karlsruhe, Germany) served as control Virus. NRVCM were infected with a multitude of infection (moi/ifu) of 50 moi, or otherwise indicated. Similarly, for the generation of expression plasmids, respective genes cloned into pDonR221 vector were shuttled into pcDNA-Dest40 or pcDNA3.2, both Gateway expression vectors for mammalian cells.

Antibodies used for immunoprecipitation,immunofluorescence and immunoblotting

α-actinin, mouse monoclonal, Sigma-Aldrich (1:400); SUMO2/3, rabbit monoclonal, Cell signaling (1:1000); SUMO2+3, mouse monoclonal, Abcam, (1:100); Rcan1-4, rabbit serum (1:500); calcineurin A , mouse monoclonal, BD Bioscience (1:250); α-Tubulin, mouse monoclonal, Sigma-Aldrich (1:5000); β-actin, mouse polyclonal, Sigma-Aldrich (1:20000). In brackets are the utilized dilution factors for all applications, unless stated otherwise.

Protein preparation

NRVCM were lysed by 3 freeze-thaw cycles in lysis buffer containing 20 mM Tris , 10 mM DTT, 500mM Sodium chloride, 1% NP40, 12,5% Glycerol and phosphatase- and protease inhibitor cocktails. Cell debris was removed by centrifugation at 12,000 x g for 20 minutes and protein concentration was determined photometrically by Biorad DC-assay method (Biorad, Munich, Germany).

Subcellular fractionation

NRVCM were fractionated using the Subcellular Protein Fractionation Kit for Cultured Cells from ThermoFisher according to the manufacturer’s instructions. In brief, cells were pelleted and re-suspended in cytoplasmic-, membrane- and nuclear lysis buffers consecutively, harvesting each fraction with the appropriate buffer. Method-integrity was verified by controlling the fractions for Histone H3 (Cell signalling, #4499) and GAPDH (Sigma, #G8795) proteins.

Immunoblotting

Protein samples were resolved by 10% SDS-PAGE, transferred to a polyvinylidenefluoride membrane and immunoblotted. After two hours of blocking in 5% dry-milk in TBS-T, Primary antibody was applied

overnight at 4 °C followed by incubation with a suitable HRP-coupled secondary antibody (1:10000) (Santa Cruz, Heidelberg, Germany). Then, signals were visualized using ECL-select chemiluminescence kit (GE Healthcare, Freiburg, Germany) and detected on Fluorchem Q imaging system (Biozym, Hessisch Oldendorf, Germany). Quantitative densitometric analysis was carried out with ImageJ/Fiji version 1.46.

RNA isolation and quantitative real-time PCR

Total RNA was isolated from cultured cells using QIAzol lysis reagent (Qiagen, Germany) following the manufacturer's instructions. One µg of DNA-free total RNA was transcribed into cDNA using the Superscript III first strand cDNA synthesis kit (Life Technologies, Darmstadt, Germany). For qRT-PCR, the EXPRESS SYBR GreenER Reagent (Life Technologies, Darmstadt, Germany) was used in CFX96 real-time Cycler (Biorad, Munich, Germany). Cycling conditions are: 3 minutes at 95°C, followed by 40 cycles of (15 seconds at 95°C, and 45 seconds at 60°C, a common step for annealing and extension at which step the data was collected). Rpl32 was used as an internal normalization control [1] using the delta-delta-Ct algorithm. All the experiments were performed in quadruplicate or hexaplicate and repeated two or three times. A list of the utilized qRT-PCR and Multiplex-PCR primers can be found in Table S 1.

Reporter gene assays

All the reporter gene assays shown in this work were performed either in NRVCM or C2C12-myoblasts. Cells were infected with combinations of different viruses expressing SUMO2 (50 moi), SUMO2ΔGG (50 moi), ΔCnA (50 moi), and LacZ as control or a filler virus to maintain equal virus load, along with adenovirus NFAT-reporter-luc (10 moi) carrying a firefly luciferase and AdRen-luc carrying renilla luciferase (5 moi, for normalization of the measurements). For SUMO2 knockdown experiments, NRVCM were Infected with an Adenovirus containing a synthetic microRNA against SUMO2 or a non-targeting control micro RNA termed miR Neg. C2C12 cells were transfected with siRNA against SUMO2 (Ambion, #4390816) or a control-siRNA (control siRNA-A, Santa Cruz). Studies were performed using a dual luciferase reporter assay (Promega, Mannheim, Germany) according to the manufacturer's instructions. Chemiluminescence was detected photometrically on infinite m200 PRO system (Tecan,

Groedig, Austria). All luciferase experiments were performed in quadruplicate or hexaplicate and repeated two or more times.

Immunoprecipitation of endogenous proteins

Mouse left ventricles were homogenized using an Ultra-turrax tissue separator in RIPA lysis buffer containing 50mM Tris-HCl (pH 7.5), 150mM NaCl, 1% NP40, 0.5% Na-Deoxycholate, 0,2% SDS, whereas, C2C12-Cells were lysed with 3 freeze-thaw cycles in Native lysis buffer (NLB) containing 50 mM Tris-HCL (pH 7.5), 150 mM NaCl, 1 mM EDTA and 5% Glycerole (v/v). Approximately 4 µg of anti-SUMO2+3 antibody (mouse monoclonal, Abcam) or anti-HA antibody (mouse monoclonal, Sigma) was allowed to interact with 1 mg protein in total volume of 1 mL (in 1.5 mL reaction tubes) for 6 h at 4 °C, to which, 50 µl of equilibrated Dynabeads (Life Technologies, Darmstadt, Germany) were pipetted and and allowed IgG-bound proteins to bind to the beads overnight at 4 °C. Protein lysate was removed after placing microcentrifuge tubes on magnetic stand. Beads were then washed for 6 times with native lysis buffer. Precipitated proteins were eluted with 50 µl of 2x Laemmli buffer, 10-20 µl of which was subjected to SDS-PAGE, followed by transfer to polyvinylidenefluoride membranes and immunoblotted using monoclonal anti-calcineurin A antibody (mouse monoclonal, BD Bioscience). Co-IP experiments were performed twice or more.

Immunofluorescence microscopy

Cell size measurement was carried out in NRVCM which were prepared as described [2, 3]. Cells were seeded on collagen-coated glass-coverslips and grown in 10% serum containing DMEM-Medium with Glucose, L-Gln and Pen/Strep. NRVCMs were fixed with 4% paraformaldehyde for 10 min at room tepmperature, permeabilized, washed and blocked with 0.1% Triton X-100 in 2.5% BSA in PBS for one hour at room temperature. Cells were then incubated for 90 minutes with the primary antibodies using the following dilutions: monoclonal mouse anti-α-actinin (Sigma, 1:400), Respective secondary antibodies conjugated with AlexaFluor546 (Invitrogen) were incubated for one hour at a dilution of 1:1000 in 2.5%BSA in PBS containing DAPI (4',6'-diamidino-2-phenylindole). Fluoromount (Biozol, Eching, Germany) was used for mounting. Fluorescence images were taken with a Keyence BZ-9000 fluorescence

microscope with a 20x CFI Plan Apo λ lens with an NA of 0.75 at room temperature. Images taken with the in-built CCD-camera were processed and analyzed by BZ-II Analyzer (Keyence)

Cell surface area measurements

Cell size measurements were carried out as described [4]. In brief, 5x5x5 (x y z) pictures were taken in 20x magnification and merged. The cell size was measured using Keyence's HybridCellCount software module in fluorescence intensity single-extraction mode. First, fluorescence-intensity thresholds were set for a reference picture. Therefore α-actinin whole-cell staining was set as the target area and the DAPI-stained nuclei were then extracted from each target area in order to determine the number of nuclei per target area. The data set's normal distribution was tested by utilized the Kolmogorow-Smirnow-test. Given a failed normality test we have applied the Wilcoxon rank-sum test (two groups, one variable) or the non-parametric Friedman test (two-way ANOVA by ranks).

Proximity ligation assay

Proximity-ligation was performed as described by the manufacturer (DuoLink InSitu Proximity-Ligation). In brief, fixed cells were blocked in 2.5 % BSA in PBS and after three washing steps incubated with two primary antibodies against SUMO2/3 (ms, Abcam, 1:1000) and CnA (rb, Upstate, 1:50) over night at 4 °C. Negative control-condition contained no primary antibody. After washing the cells, incubation with the two different species-specific PLA-probes occurred (minus and plus strands) for 1h at 37 °C in the dark. Probes were then annealed and ligated for 30 minutes at 37 °C in the dark, followed by the amplification step for close-proximity probes. This step also labels amplified areas with a fluorophore emitting light at 527 nm. Primary antibodies that are in closer proximity than 30-40 nm yield green dots and indicate direct protein-protein interaction. Utilizing the Keyence Hybrid Cell Count module, first, DAPI-stained nuclei were outlined using fluorescence threshold cutoff and afterwards, the fluorescence emitted from PLA-staining dye was extracted from the nuclei to calculate the percentage of "positive" nuclei.

Generation of AAV9-vectors

The cDNA sequences of SUMO2 and SUMO2ΔGG were cloned into a single-stranded AAV vector backbone (pSSV9-CMV-MLC1500-luc) with Primers listed in Table S 1. In Brief: ORF-PCR was performed and samples were XbaI-digested. After ligation of the digested ORF amplicon into the AAV genome plasmid (digested with the same enzyme), correct fragment size and orientation were controlled by agarose gel-electrophoresis and sequencing, resulting in pSSV9-CMV-MLC1500-SUMO2 or -SUMO2ΔGG. AAV9 vectors were generated by co-transfection of helper plasmid pDP9rs and either pSSV9-CMV-MLC1500-SUMO2, -SUMO2ΔGG or -luciferase (control). AAV9 vectors were then purified using iodixanol step gradient ultracentrifugation as described previously [5-7]. Genomic titers were determined by quantitative by qRT-PCR.

AAV9 mediated gene-transfer

Animals were injected with up to 10^{12} vector genomes per animal in a total of 120µL PBS into the tail-vein of 8 week old mice. Six weeks later, the animals were sacrificed after echocardiography to harvest the organs.

Echocardiography

Echocardiographic analyses were carried out using a VisualSonics Vevo 1100 high frequency ultrasound system at 14 weeks of age (end point). In brief, mice were anesthetized using Isoflurane (2.5ppm) and placed onto a warming pad with continuous temperature management. EF measurements were carried out by LV trace protocol comparing enddiastolic and endsystolic LV Volume. The following M-mode based parameters were obtained in a short axis on the level of the papillary muscles: interventricular septum and posterior wall thickness, as well as left ventricular fractional shortening. {FS% = [(LVEDD-LVESD)/LVEDD]*100}. Analyses were obtained by a genotype-blinded examiner. Echocardiographic data is summarized in.

Isolation and culture of adult rat ventricular cardiomyocytes (ARVCMs).

Adult rat cardiomyocytes (ARVCM) were isolated from Wistar rats as described[2, 8]. Animals were anaesthetised with sodium pentobarbital (50 mg per kg, intraperitoneal), the aorta cannulated after excision of the hearts and perfused with a rate of 12 mL/min in a Langendorff perfusor. Hearts were first

perfused with Ca^{2+}-free medium (ACM) under a 95% O2/5%CO2 equilibrium. After 5 min the perfusion was changed to ACM containing collagenase (0.5Uml1, type A; Roche) for 20 min. Finally, the hearts were perfused with low Na^{+}, high sucrose Tyrode solution for 15 min. Then, left ventricles of the digested hearts were cut into small pieces and cells dissociated by careful agitation. The cells were resuspended in ACM without BDM containing 2mM calcium that was gradually reintroduced at 25°C. Cardiomyocytes were plated with a density of 40,000 cells per cm^2 on laminin-coated dishes, followed by adenoviral infection, 4 h after seeding of the cells. ARVCM were cultured for 24 h after infection. AdLacZ, AdS2, AdS2ΔGG, AdΔCnA: 1000 moi; AdNFAT-RE-Firefly-Luc, AdRenilla-Luc: 400 moi.

Human heart samples

The human study was approved by the local ethics committee with informed consent from patients, and conforms to the declaration of Helsinki.

Supplementary Figures

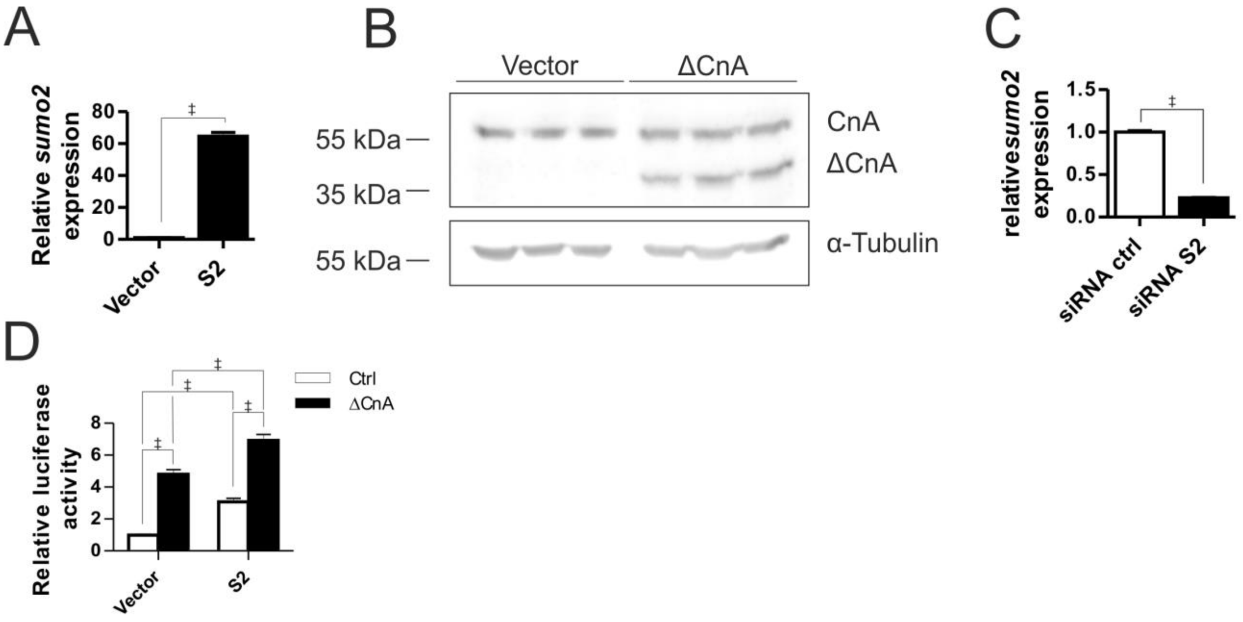

Figure S 1: Expression of SUMO2 and ΔCnA in C2C12 cells. A, mRNA expression of *sumo2* in *sumo2* transfected cells compared to control. B, western blot showing ΔCnA overexpression. C, mRNA expression of *sumo2* in the presence of siRNA against *sumo2* compared to control siRNA. A-C were performed in C2C12 cells stably expressing NFAT-RE driven firefly luciferase in hexaplicates. D, NFAT-RE firefly luciferase construct was expressed via transfection of mammalian expression plasmids and luciferase activity was measured in the presence of either empty vector or S2 in the presence or absence of ΔCnA. Two independent experiments in hexaplicates were performed Statistical calculations were carried out by two-tailed Student's t-test (A, C) or Two-Way-ANOVA (D) *: $p<0.05$, †: $p<0.01$, ‡: $p<0.001$.

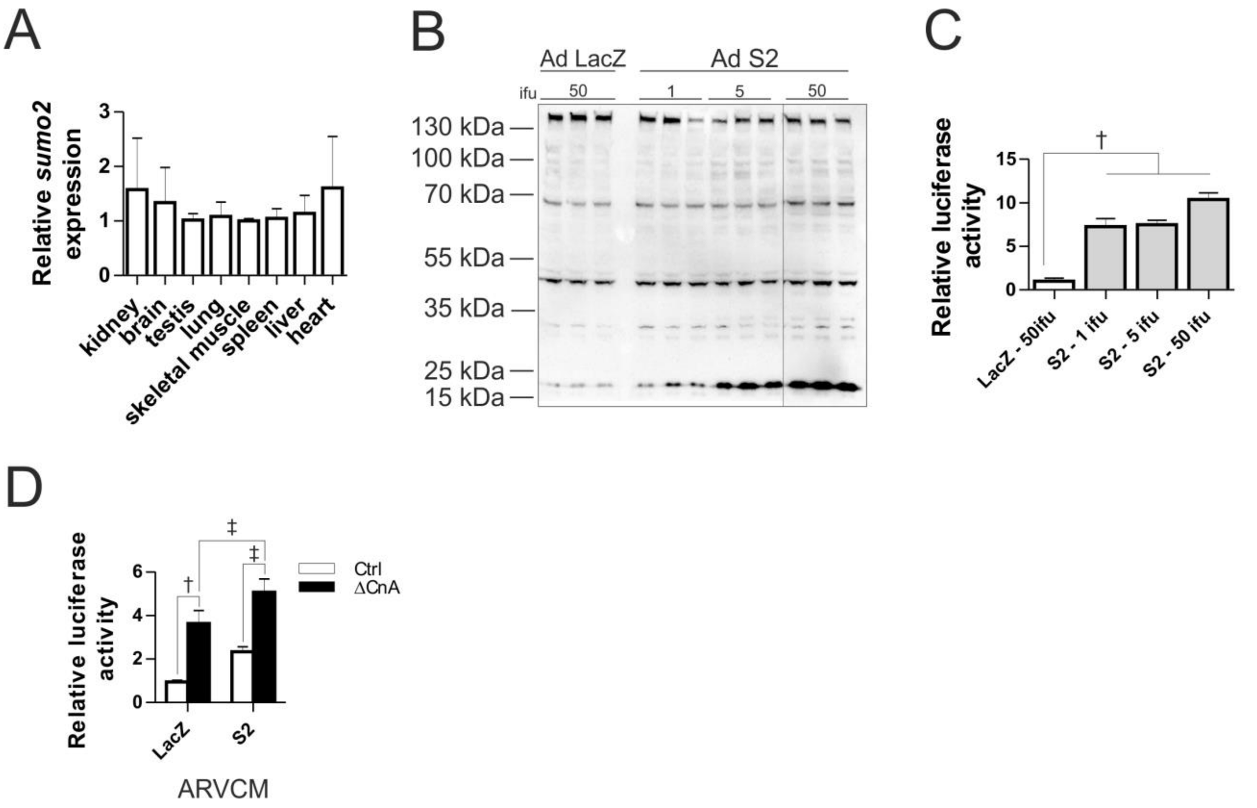

Figure S 2: Tissue distribution of endogenous *sumo2* mRNA in mouse and its dose dependent effects in NRVCMs. A, Expression profile of *sumo2* in different mouse tissues determined by qRT-PCR. B, Dose-curve western blot analysis for the adenoviral overexpression of S2 in NRVCM. Dividing line represents rearrangements of respective lanes within one membrane. C, NFAT-RE firefly luciferase construct was expressed via adenoviral infection and luciferase activity measured in the presence or absence of different amounts of S2. D, NFAT-RE firefly luciferase construct was expressed via adenoviral infection and luciferase activity was measured in the presence of either LacZ or S2 in the presence or absence of ΔCnA. Three independent experiments in hexaplicates were performed. Statistical calculations were carried out by One-Way-ANOVA (B) or Two-Way-ANOVA (C), †: $p<0.01$, ‡: $p<0.001$.

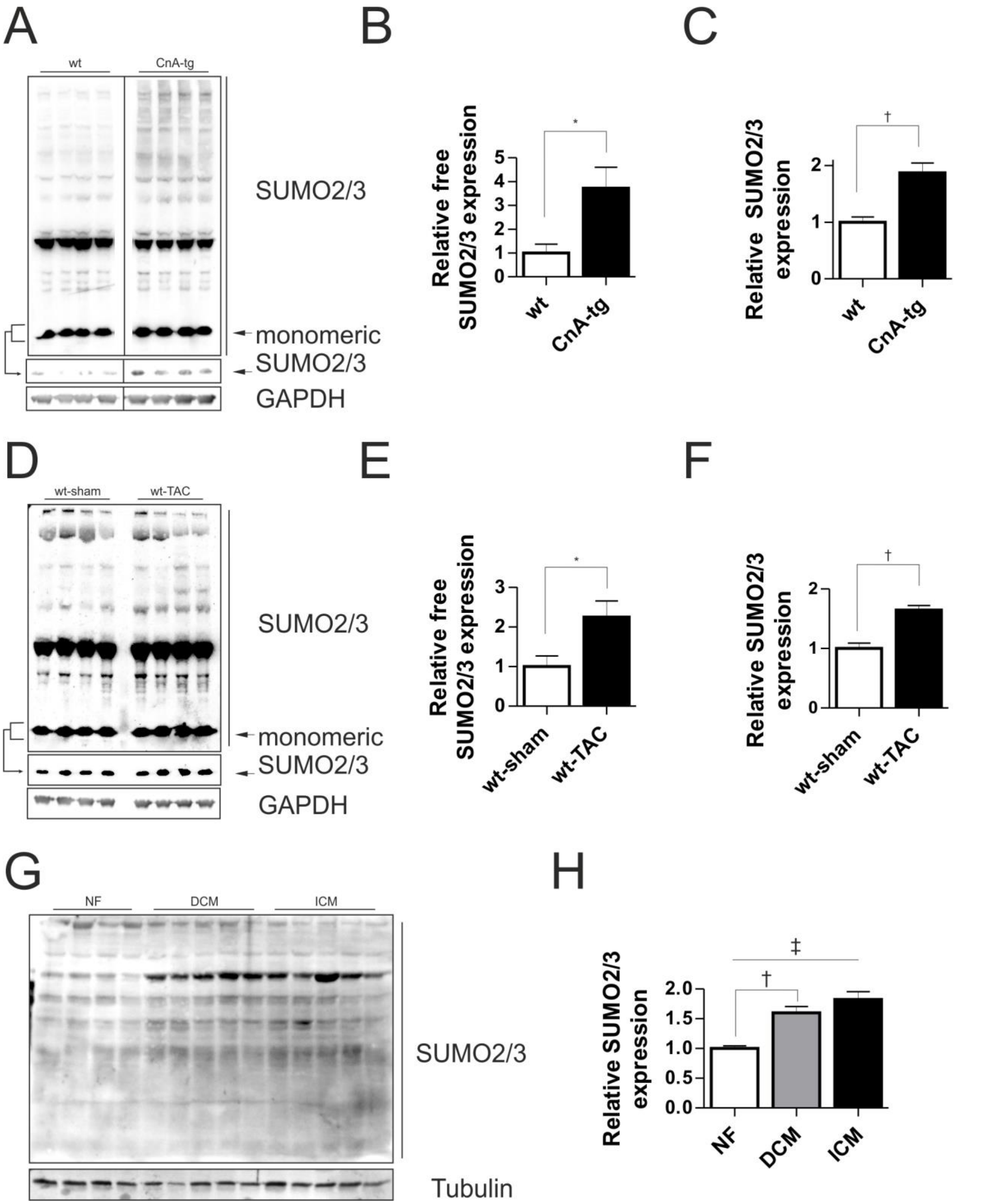

Figure S 3: S2 expression and sumoylation is increased in disease models of pressure overload and hypertrophy. A, western blot showing S2 expression in wildtype (wt) and calcineurin transgenic mice

(age: 18 week); middle-panel shows a shorter exposure band of monomeric SUMO2/3. Relative expression of monomeric SUMO2/3 (B), and total SUMO2/3 (C) was calculated by densitometric analysis using GAPDH as an endogenous control. D, western blot showing S2 expression in sham and TAC operated mice (age: 8 weeks old, following 4 weeks of TAC); middle-panel shows a shorter exposure band of monomeric SUMO2/3. Relative expression of monomeric SUMO2/3 (E), and total SUMO2/3 (F) was calculated by densitometric analysis using GAPDH as an endogenous control. N= 4 mice per group. G, western blot showing S2 expression in human heart samples fom non-failing (NF), dilated-cardiomyopathy (DCM) and ischemic cardiomyopathy (ICM) patients. H, relative densitometric analysis using Tubulin as endogenous control, n=4 (NF) or n=5 (DCM, ICM). Statistical calculations were carried out by One-Way-ANOVA (B, H) or Two-Way-ANOVA (C), *: $p<0.05$, †: $p<0.01$, ‡: $p<0.001$. Dividing line represents rearrangements of respective lanes within one membrane.

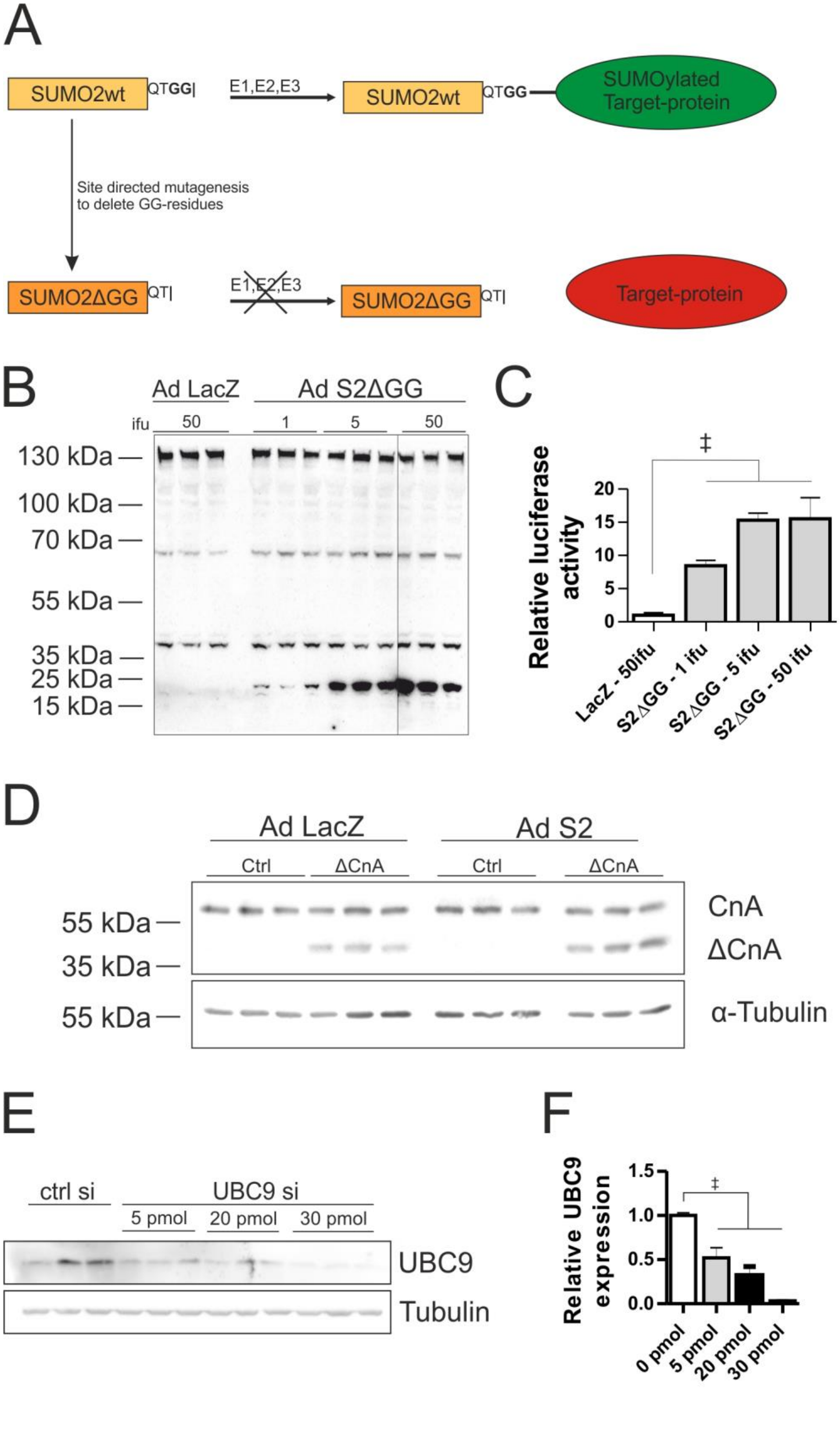
A
SUMO2wt
QTGG|
E1,E2,E3
SUMO2wt
QTGG
SUMOylated Target-protein
Site directed mutagenesis to delete GG-residues
SUMO2ΔGG
QT|
E1,E2,E3
SUMO2ΔGG
QT|
Target-protein
B
Ad LacZ
Ad S2ΔGG
ifu
50
1
5
50
130 kDa
100 kDa
70 kDa
55 kDa
35 kDa
25 kDa
15 kDa
C
‡
Relative luciferase activity
20
15
10
5
0
LacZ - 50ifu
S2ΔGG - 1 ifu
S2ΔGG - 5 ifu
S2ΔGG - 50 ifu
D
Ad LacZ
Ad S2
Ctrl
ΔCnA
Ctrl
ΔCnA
55 kDa
35 kDa
55 kDa
CnA
ΔCnA
α-Tubulin
E
ctrl si
UBC9 si
5 pmol
20 pmol
30 pmol
UBC9
Tubulin
F
‡
Relative UBC9 expression
1.5
1.0
0.5
0.0
0 pmol
5 pmol
20 pmol
30 pmol

Figure S 4: S2ΔGG mutagenesis, strategy and UBC9-knockdown. A, schematical drawing of the mutagenesis strategy. B, Dose-curve western blot analysis for the adenoviral overexpression of S2ΔGG overexpression in NRVCM. C, NFAT-RE firefly luciferase construct was expressed via adenoviral infection and luciferase activity measured in the presence or absence of different amounts of S2ΔGG**.** D, Western blot showing overexpression of ΔCnA in the presence or absence of overexpressed S2 compared to LacZ control. E, western blot showing dose-curve for the UBC9-siRNA and F, respective densitometric analysis. Three independent experiments in hexaplicates (Luciferase) or triplicates (western-blot) were performed. Statistical calculations were carried out by One-Way-ANOVA ‡: p<0.001.

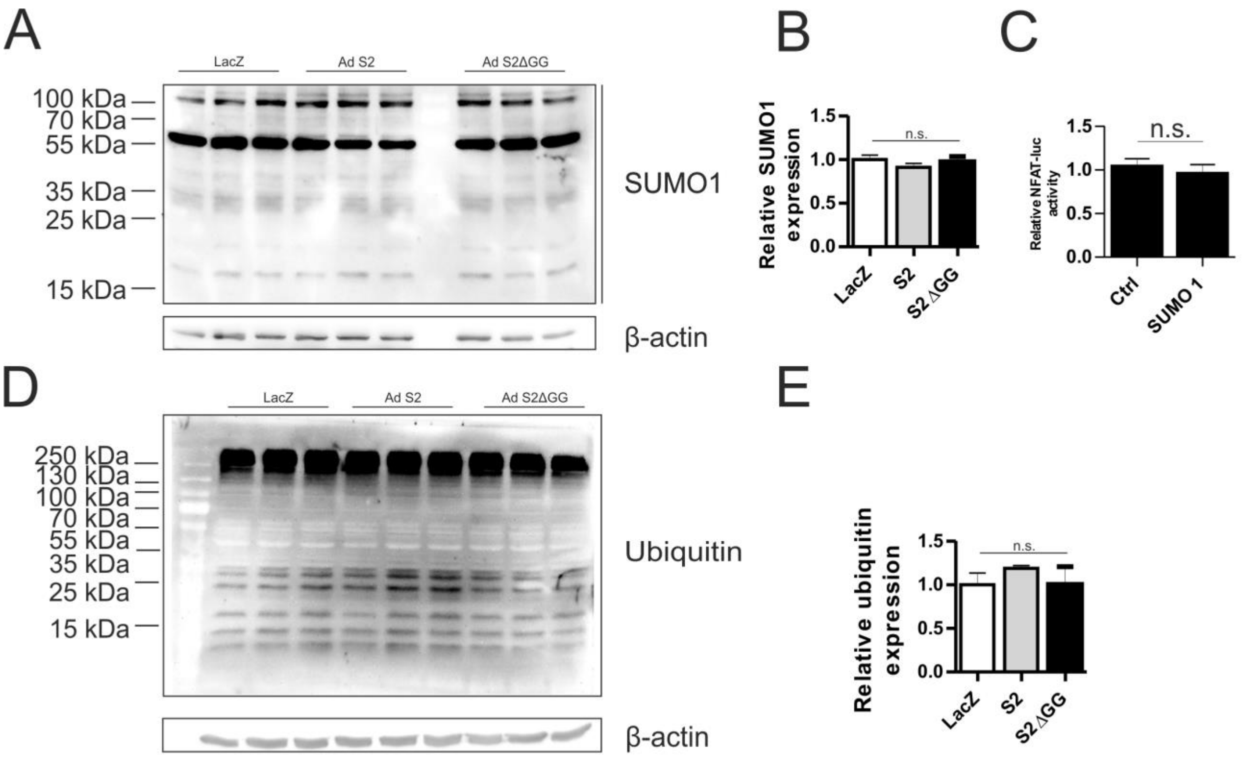

Figure S 5: SUMO1 and Ubiquitin expression profiles. A, western blot showing the expression of SUMO1 with overexpressed S2 or S2ΔGG compared to LacZ control with B, respective densitometric analysis. C, NFAT-RE firefly luciferase construct was expressed via adenoviral infection and luciferase activity measured in the presence or absence of SUMO1 (S1). Shown are the mean of three independent

experiments in triplicates. D, Western blot showing the expression of ubiquitinated proteins whith overexpressed S2 or S2ΔGG compared to LacZ control with E, respective densitometric analysis. Statistical calculations were carried out by ttest (B) or One-Way-ANOVA (B, E)

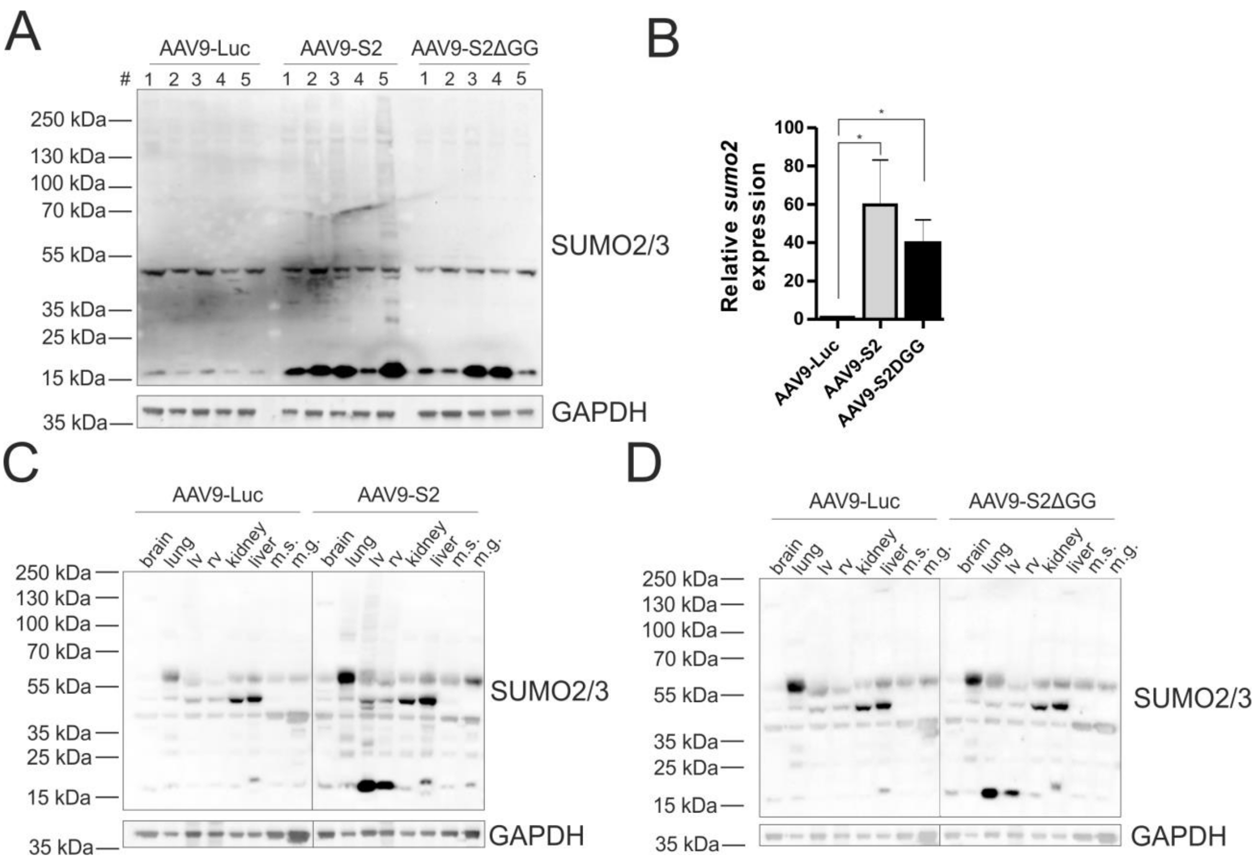

Figure S 6: AAV9-SUMO2-ovexpression and tissue-distribution in mice. A, western blot showing SUMO2/3 expression in individual left-ventricle protein-lysates of the mice. B, mRNA expression of S2 and S2ΔGG. C, D, multi-tissue expression of S2 in AAV9-S2 (C) or AAV9-S2ΔGG (D) injected mice. Organs for these protein lysates came from the highest-expressing mice, i.e. mouse #5 from AAV9-S2 and mouse #3 from AAV9-S2ΔGG; lv= left ventricle, rv= right ventricle, m.s. = musculus soleus, m.g. = musculus gastrocnemicus. Statistical calculations were carried out by One-way ANOVA with Student-Newman-Keuls post-hoc test (A-J). *: $p<0.05$.

Supplementary Tables

Table S 1: Primers used for the cloning of different mouse SUMO2 constructs and qRT-PCR primers.

**Primer Name**	**Sequence 5' > 3'**	**Purpose**
S2_OS_F	GGGGACAAGTTTGTACAAAAAAGCTGGCACCATGGCC GACGAAAAGCCCAAGG	Full length S2 with tag
S2_OS_R	GGGGACCACTTTGTACAAGAAAGCTGGGTCGCCACCG TCTGCTGTTGGAA	Full length with tag
S2ΔGG_R	GGGGACCACTTTGTACAAGAAAGCTGGGTCGCCTCGC CTTCAGTAGACCGTCTGCTGTTGGAA	Non-conjugatable S2
XbaI_S2_F	GCCTCTAGAGCCACCATGGCCGACGAAAAGCCCAAGG	AAV9-generation of S2
XbaI_S2_R	GCCTCTAGATCAGTAGACACCTCCCGTCTGCTG	AAV9-generation of S2
XbaI_S2ΔGG_R	GCCTCTAGATCAGTAGACCGTCTGCTGTTGGAA	AAV9-generation of non-conjugatable S2
qRT-huS2_F	CCGATTTGACGGGCAACCAATCA	qRT-Primers for human *sumo2*
qRT-huS2_R	ACACCTCCCGTCTGCTGTTGGA	
qRT-mm_r_S2_F	ATTGTGAACGGCAGGGTTTG	qRT-Primers for mouse / rat *sumo2*
qRT-mm_r_S2_R	GTCTGCTGCTGGAACACATC	
Rpl32_F	CTGCTGATGTGCAACAAATCT	qRT-Primers for *rpl32* (housekeeping / control)
Rpl32_R	GCTGTGCTGCTCTTTCTACAAT	
Nppa_F	GGAGCAAATCCTGTGTACAGTG	Multiplex Primer and probe labeled with dye and quencher for *Nppa*
Nppa_R	ACCTCATCTTCTACCGGCAT	
Nppa_probe	FAM-TGATGGATTTCAAGAACCTGCTAGACCA-BHQ1	
Nppb_F	ACAAGATAGACCGGATCGGA	Multiplex Primer and probe labeled with dye and quencher for *Nppb*
Nppb_R	AGCCAGGAGGTCTTCCTACA	
Nppb_probe	HEX-TCAGTGCGTTACAGCCCAAACGA-BHQ1	
Rpl32_F	CTGCTGATGTGCAACAAATCT	Multiplex Primer and probe labeled with dye and quencher for *rpl32* (housekeeping / control)
Rpl32_R	GCTGTGCTGCTCTTTCTACAAT	
Rpl32_probe	Texas Red-ACTGTGCTGAGATTGCTCACAATGTGT-BHQ2	
Rcan1_fw	TAGCTCCCTGATTGCTTGTG	Multiplex Primer and probe labeled with dye and quencher for *rcan1-4*
Rcan1_rev	GGATTCAAATTTGGCCCTGG	
Rcan1_prb	Cy5.5-ACGATGATGTCTTCAGCGAAAGTGAGAC-Eclipse	

Table S2: Summary of echocardiographic data. Shown are ejection fraction (EF) and fractional shortening (FS) in %, wall-dimensions in mm. All data obtained in the presence of AAV9-mediated overexpression of S2 or S2ΔGG, compared to Luciferase control. Mean of n=5 mince per group, 14 weeks old, injection of AAV9 at 8 weeks. Statistical calculations were carried out by one-way-ANOVA.

**Parameter**	**AAV9_Luc**	**AAV9_S2**	**AAV9_S2ΔGG**	**p-value (One-way-ANOVA)**
EF (LV Trace)	37.149034	26.722976	30.320452	**0.0045**
FS	25.121406	19.130006	20.317126	**0.0297**
IVS;d	0.6222412	0.7337932	0.7165518	0.0748
IVS;s	0.8793106	1.0030172	0.9013794	0.2754
LVID;d	4.2389654	4.3024138	4.1767242	0.8748
LVID;s	3.2687932	3.477069	3.1002586	0.3372
LVPW;d	0.7393104	0.7974138	0.7730172	0.1748
LVPW;s	0.9962068	0.913707	1.077931	0.1405

Supplementary Videos

Representative M-Mode videos of the heart for AAV9-mediated gene transfer in mice, acquired as explained in Methods.

1. MMode_AAV9_Luc.avi

2. MMode_AAV9_S2.avi

3. MMode_AAV9_S2dGG.avi

Full blots for cropped western blot panels.

Fig. 1B, E, 2G, 4A: membranes were cut out prior to antibody-incubations and non-cropped.

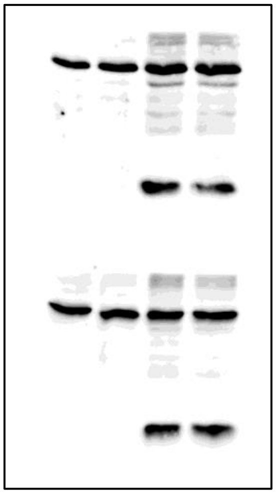

Fig. 2A: Full length blot that was cropped to figure.

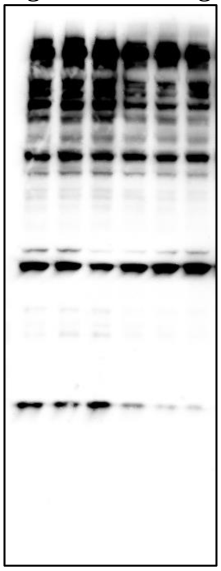

Fig. 2D: Full length blot that was cropped to figure.

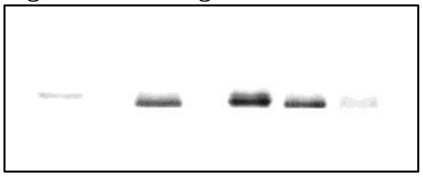

Fig. 6D: Full membrane part (upper panel)

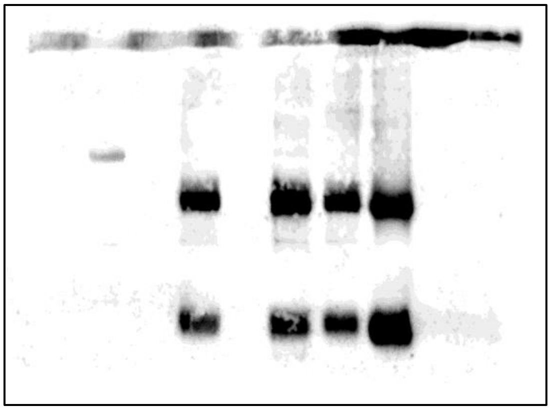

Fig. 6D: Full length blot (lower panel)

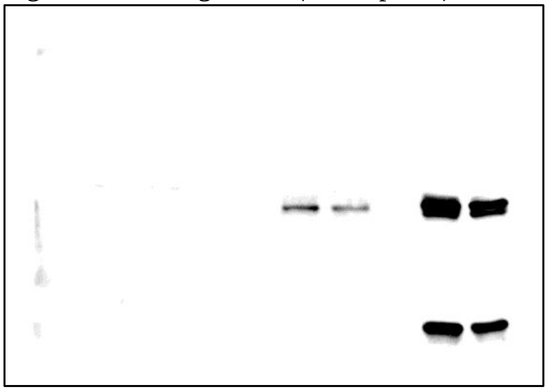

Fig. 7A: Full length blots Panel “Nuclear extract / CnA", Lanes 1-3, 7-9, 13-15 and 20 respectively.

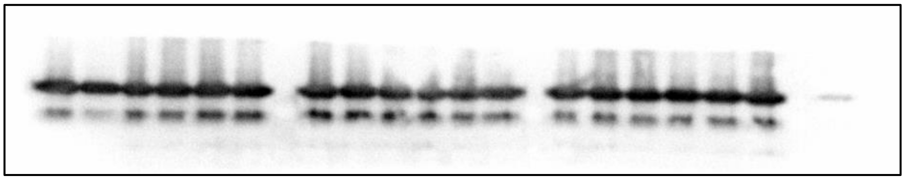

Fig. 7A: Full membrane part Panel “Nuclear extract / Histon H3", Lanes 1-3, 7-9, 13-15 and 20 respectively.

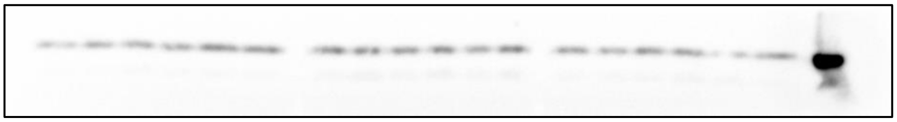

Fig. 7A: Full membrane part Panel “Nuclear extract / GAPDH", Lanes 1-3, 7-9, 13-15 and 20 respectively.

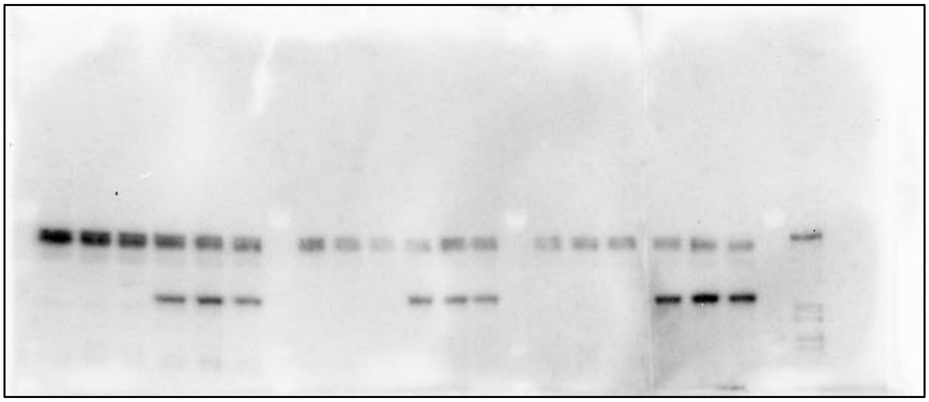

Fig. 7A: Full length blots Panel “Cytoplasmic extract / CnA", Lanes 1-3, 7-9, 13-15 and 20 respectively.

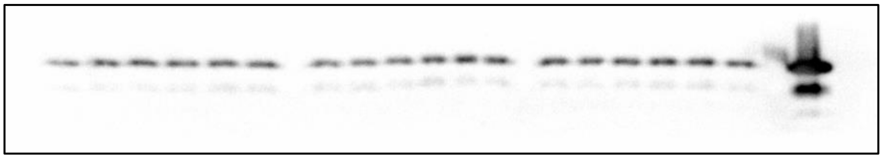

Fig. 7A: Full membrane part Panel “Cytoplasmic extract / Histon H3", Lanes 1-3, 7-9, 13-15 and 20 respectively.

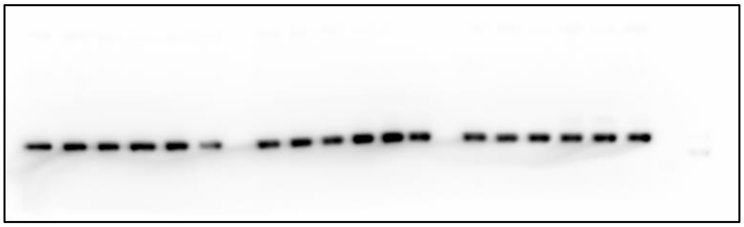

Fig. 7A: Full membrane part Panel “Cytoplasmic extract / GAPDH", Lanes 1-3, 7-9, 13-15 and 20 respectively.

Supplementary References

1. Frank D, Kuhn C, Brors B, Hanselmann C, Ludde M, Katus HA, Frey N. Gene expression pattern in biomechanically stretched cardiomyocytes: Evidence for a stretch-specific gene program. *Hypertension*. 2008;51:309-318

2. Boerries M, Most P, Gledhill JR, Walker JE, Katus HA, Koch WJ, Aebi U, Schoenenberger CA. Ca2+ -dependent interaction of s100a1 with f1-atpase leads to an increased atp content in cardiomyocytes. *Molecular and cellular biology*. 2007;27:4365-4373

3. Seeger TS, Frank D, Rohr C, Will R, Just S, Grund C, Lyon R, Luedde M, Koegl M, Sheikh F, Rottbauer W, Franke WW, Katus HA, Olson EN, Frey N. Myozap, a novel intercalated disc protein, activates serum response factor-dependent signaling and is required to maintain cardiac function in vivo. *Circ Res*. 2010;106:880-890

4. Rangrez AY, Bernt A, Poyanmehr R, Harazin V, Boomgaarden I, Kuhn C, Rohrbeck A, Frank D, Frey N. Dysbindin is a potent inducer of rhoa-srf-mediated cardiomyocyte hypertrophy. *The Journal of cell biology*. 2013;203:643-656

5. Goehringer C, Rutschow D, Bauer R, Schinkel S, Weichenhan D, Bekeredjian R, Straub V, Kleinschmidt JA, Katus HA, Muller OJ. Prevention of cardiomyopathy in delta-sarcoglycan knockout mice after systemic transfer of targeted adeno-associated viral vectors. *Cardiovascular research*. 2009;82:404-410

6. Grieger JC, Choi VW, Samulski RJ. Production and characterization of adeno-associated viral vectors. *Nature protocols*. 2006;1:1412-1428

7. Schinkel S, Bauer R, Bekeredjian R, Stucka R, Rutschow D, Lochmuller H, Kleinschmidt JA, Katus HA, Muller OJ. Long-term preservation of cardiac structure and function after adeno-associated virus serotype 9-mediated microdystrophin gene transfer in mdx mice. *Human gene therapy*. 2012;23:566-575

8. Luedde M, Flogel U, Knorr M, Grundt C, Hippe HJ, Brors B, Frank D, Haselmann U, Antony C, Voelkers M, Schrader J, Most P, Lemmer B, Katus HA, Frey N. Decreased contractility due to energy deprivation in a transgenic rat model of hypertrophic cardiomyopathy. *J Mol Med (Berl)*. 2009;87:411-422
